# PTBP1-mediated regulation of AXL mRNA stability plays a role in lung tumorigenesis

**DOI:** 10.1038/s41598-019-53097-2

**Published:** 2019-11-15

**Authors:** Chun-Yu Cho, Shih-Ying Chung, Shankung Lin, Jhy-Shrian Huang, Yen-Lin Chen, Shih-Sheng Jiang, Li-Chun Cheng, Tsu-Hsiang Kuo, Jong-Ding Lay, Ya-Yu Yang, Gi-Ming Lai, Shuang-En Chuang

**Affiliations:** 1 National Institute of Cancer Research, National Health Research Institutes, Miaoli, Taiwan; 2 Inflammation Research & Drug Development Center, Changhua Christian Hospital, Changhua, Taiwan; 3 Department of Pediatrics, Kaohsiung Medical University Hospital, Kaohsiung, Taiwan; 4 Department of Pathology, Cardinal Tien Hospital, School of Medicine, Fu-Jen Catholic University, New Taipei City, Taiwan; 5 Graduate Institute of Life Sciences, National Defense Medical Center, Taipei, Taiwan; 6 Department of Nursing, National Taichung University of Science and Technology, Taichung, Taiwan; 7 Comprehensive Cancer Center, Taipei Medical University, Taipei, Taiwan; 8 Cancer Center, Wan Fang Hospital, Taipei Medical University, Taipei, Taiwan

**Keywords:** Non-small-cell lung cancer, Cell growth, Transcription

## Abstract

AXL is expressed in many types of cancer and promotes cancer cell survival, metastasis and drug resistance. Here, we focus on identifying modulators that regulate AXL at the mRNA level. We have previously observed that the AXL promoter activity is inversely correlated with the AXL expression levels, suggesting that post-transcriptional mechanisms exist that down-regulate the expression of AXL mRNA. Here we show that the RNA binding protein PTBP1 (polypyrimidine tract-binding protein) directly targets the 5′-UTR of AXL mRNA *in vitro* and *in vivo*. Moreover, we also demonstrate that PTBP1, but not PTBP2, inhibits the expression of AXL mRNA and the RNA recognition motif 1 (RRM1) of PTBP1 is crucial for this interaction. To clarify how PTBP1 regulates AXL expression at the mRNA level, we found that, while the transcription rate of AXL was not significantly different, PTBP1 decreased the stability of AXL mRNA. In addition, over-expression of AXL may counteract the PTBP1-mediated apoptosis. Knock-down of PTBP1 expression could enhance tumor growth in animal models. Finally, PTBP1 was found to be negatively correlated with AXL expression in lung tumor tissues in Oncomine datasets and in tissue micro-array (TMA) analysis. In conclusion, we have identified a molecular mechanism of AXL expression regulation by PTBP1 through controlling the AXL mRNA stability. These findings may represent new thoughts alternative to current approaches that directly inhibit AXL signaling and may eventually help to develop novel therapeutics to avoid cancer metastasis and drug resistance.

## Introduction

Lung cancer accounts for about 10–20% of total cancer deaths. Non-small cell lung cancer (NSCLC) causes 1.4 million cancer deaths worldwide every year. Among the stage I and II patients receiving potentially curative resection, five-year survival ranges from 25 to 45%. Patients with more advanced diseases of stage IIIa and IIIb have a five-year survival of 15 and 5%, respectively. The conventional cytotoxic chemotherapy has a limited efficacy for this disease. The discovery of mutations in the EGFR gene (in 15–30% of affected individuals worldwide) that may sensitize tumors to EGFR-tyrosine kinase inhibitors (TKIs), such as erlotinib, is astonishing, but the response is not durable. Among other mechanisms, lung tumors may also acquire resistance to TKIs via AXL over-expression in many patients^1^.

AXL is a transmembrane receptor tyrosine kinase (RTK) whose over-expression has been reported in many human cancers^2,3^ and AXL signaling promotes cell proliferation, survival, invasion and metastasis, at least in part through activation of the PI3K-AKT^4–6^ and RAS-MEK-ERK pathways^7^. Our recent study showed that NF-kB signaling pathway was involved in the AXL-associated invasiveness and drug resistance, and dual inhibition of NF-kB and AXL phosphorylation by sulfasalazine may provide an opportunity to effectively attenuate the invasiveness and drug resistance of NSCLC cells^8^. Additionally, we also found that upregulation of AXL by chemotherapy drugs might confer drug resistance in acute myeloid leukemia^9^. In lung adenocarcinoma, AXL has been shown to be associated with lymph node status and the patient’s clinical stage^2^. AXL not only is crucial in the *in vitro* invasiveness but also may play an important role in cancer progression in patients. Of note, only over-expression and/or activation of the AXL protein, but not AXL gene mutations, have been reported in cancer patients. Targeting the tyrosine kinase activity with antibodies or small molecule inhibitors has been a focus in the treatment of tumors. However, this strategy usually results in development of TKI resistance. One of the resistance developed is through the activation of AXL expression. AXL activation is a mechanism of acquired resistance to EGFR inhibitors in NSCLC and that its inhibition can restore TKI sensitivity^1,10^.

The RNA-binding protein PTBP1 was originally identified as a protein that bound to the polypyrimidine rich region within introns and was described as a regulator of splicing in the nucleus^11,12^. Beside its role in splicing, PTBP1 has also been implicated in the regulation of other aspects of RNA metabolisms^12^. Changes in RNA alternative splicing sites have been correlated with malignant transformation^13–17^. The PTBP1’s regulatory function in RNA splicing is apparently needed for tumor cell growth^14,18–20^. However, PTBP1 could export from nucleus to cytoplasm and play different roles, such as mRNA stability and cap-independent translation driven by the internal ribosomal entry site (IRES). Recent studies indicate that CD154 has anti-tumor activity and growth-inhibitory effects and that PTBP1 is able to stabilize CD154 mRNA^21,22^. PTBP1 can bind and up-regulate the IRES activity of the tumor suppressor p27 mRNA and the Apaf-1 mRNA^23,24^. PTBP1 could down regulate hypoxia-inducible factor 1α (HIF-1α) expression via regulating its mRNA stability and was able to inhibit cell invasion when localized in the cytoplasm^25^. PTBP1 was also found to induce p19 mRNA expression via promoter regulation and inhibit cell proliferation^26^. These findings suggest that PTBP1 may play different roles in the nucleus vs. cytoplasm. Altered PTBP1 expression in cancer cells has been documented^27,28^. Studies focusing on its RNA splicing regulatory role in the nucleus have found that PTBP1 may promote a malignant phenotype in cancer cells^17,29–31^.

Our recent data have demonstrated the involvement of micro-RNA in the feedback regulation of AXL in its mRNA 3′-untranslated region (3′-UTR) and is important for balanced expression and may possess therapeutic potentials^32^. While the expression levels of AXL are in accordance with the invasiveness, our study indicated that the AXL promoter activity was the opposite in trend as demonstrated in reporter assays. Importantly, the AXL expression is correlated with AXL 5′-UTR reporter activity but not 3′-UTR reporter activity. We hypothesize that there might exist stabilizing/destabilizing elements in AXL mRNA whereby its expression is regulated at the post-transcriptional level. In the present study, we clarify the involvement of PTBP1 in the regulation of AXL mRNA stability. PTBP1 directly binds the 5′-UTR of AXL mRNA *in vitro* and *in vivo* and decreased the stability of AXL mRNA. The RNA recognition motif 1 (RRM1) of PTBP1 is crucial for this interaction. In addition, ectopic overexpression of AXL may counteract the PTBP1-mediated apoptosis. Moreover, we have demonstrated that PTBP1 may inhibit tumorigenesis of lung cancer cells *in vivo*. Finally, our tissue microarray (TMA) data and the correlation analysis of Oncomine datasets both showed that PTBP1 was negatively correlated with AXL expression in lung tumor tissues. Together, these results suggest that, as an alternative to direct inhibitors of AXL, elucidation of the mechanisms underlying how AXL is regulated at the mRNA level may help develop novel AXL-targeting approaches for cancer treatment in the future.

## Results

### AXL expression positively correlates with the AXL 5′-UTR reporter activity but not the 3′-UTR reporter activity

**Figure 1:**
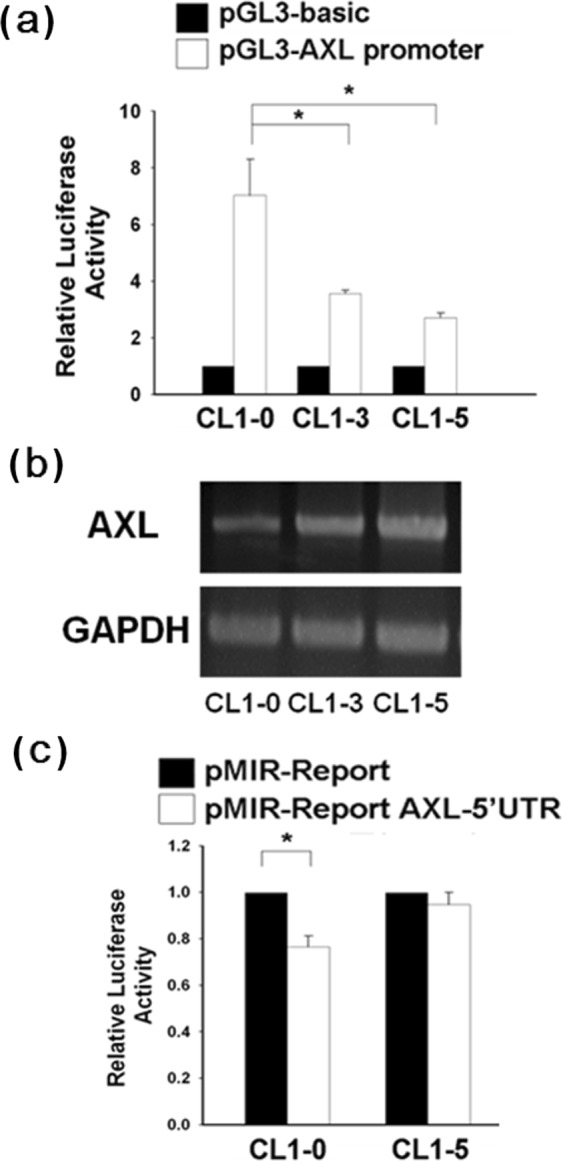
The AXL mRNA expression is correlated with AXL 5′-UTR reporter activity. (**A**) Inverse correlation between AXL mRNA level and AXL promoter reporter activity. CL1-0, CL1-3 and CL1-5 cells were transfected with the pGL3-AXL promoter luciferase construct, respectively. Luciferase reporter activity was assayed 48 h later. (**B**) The steady-state AXL mRNA levels of cell lines were compared by RT-PCR using GAPDH as an internal control. (**C**) AXL 5′-UTR reporter activity in CL1-0 and CL1-5 cells. The pMIR-Report-AXL 5′-UTR or p-MIR-Report (mock vector) plasmid was co-transfected with Renilla plasmid (serving as an internal control for transfection efficiency) into CL1-0 and CL1-5 cells. After 24 h, luciferase activity of the cell lysates was measured. Relative fold change in luciferase activity was plotted with respect to the pMIR-Report mock control. All reporter assays were performed in triplicate and the Renilla reporter activity was used to normalize the transfection efficiency. The error bars represent SD. *P < 0.05.

Since AXL plays important roles in cancer progression, it is therefore crucial to elucidate the molecular mechanisms of AXL expression regulation. We constructed the AXL promoter reporter for monitoring its promoter activities. As a result, while the AXL promoter activity was inversely correlated with the AXL mRNA levels in both lung cancer cell lines and breast cancer cell lines as determined by reporter assays (Fig. 1a,b and Supplementary Fig. 1), the AXL mRNA levels were positively correlated with its 5′-UTR reporter activity (Fig. 1c) but not its 3′-UTR reporter activity in our recent study^32^. These results indicate that expression of AXL mRNA may be regulated by certain factors through its 5′-UTR. To identify the regions of the 5′-UTR potentially mediating the regulation of AXL mRNA, we discovered a possible destabilizing element in the AXL 5′-UTR and found that the polypyrimidine tract-binding protein 1 (PTBP1) was highly likely involved in this post-transcriptional regulation by using sequence alignment and RNA structure analysis (Supplementary Fig. 2).

### PTBP1 regulates AXL mRNA expression

**Figure 2:**
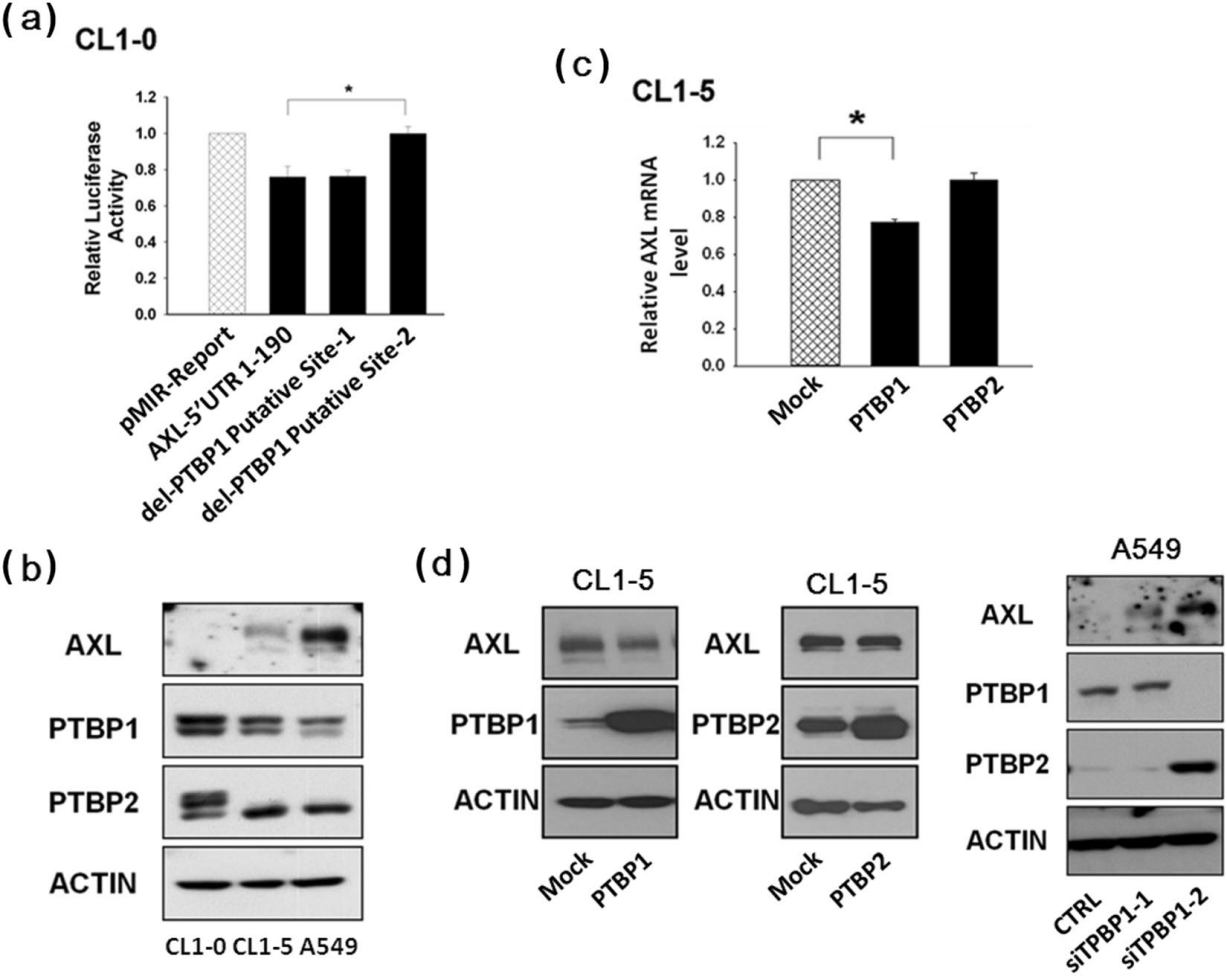
Expression of PTBP1 and its involvement in AXL regulation. (**A**) Identification of the PTBP1-binding region of AXL 5′-UTR. Luciferase reporter vectors pMIR-Report-AXL 5′-UTR (intact, lane 2) or pMIR-Report-AXL 5′-UTR (deletion mutants, lanes 3 and 4) were transfected into CL1-0 cells, and luciferase activity was measured 24 h later. (**B**) Inverse correlation between endogenous levels of AXL and PTBP1 proteins in the low invasive CL1-0 cells and the highly invasive CL1-5 and A549 cells. (**C**,**D**) Endogenous AXL mRNA and protein levels were up- and down-regulated by PTBP1 siRNA and PTBP1, respectively. In these experiments, A549 cells were transfected with PTBP1 siRNA and CL1-5 cells were transfected with PTBP1 or PTBP2. Expression levels of AXL mRNA and protein were measured, respectively, by quantitative real-time RT-PCR and Western blot 48 h after transfection. Actin was used as the internal control. The values were derived from three independent experiments. Error bars represent SD. *P < 0.05.

We performed the 5′-UTR reporter assay for monitoring AXL mRNA expression. The reporter constructs included that of the wild-type and those of various mutations in the 5′-UTR sequence. We found that deletion of the PTBP1 putative targeting site-2, but not site-1, from the AXL 5′-UTR recovered its reporter activity in CL1-0 and MDA-MB-231 cells (Fig. 2a, Supplementary Fig. 3a). We have observed an inverse correlation between endogenous levels of AXL and PTBP1 proteins when comparing the low invasive CL1-0 cells and the highly invasive CL1-5 and A549 cells. Similar results were obtained using other cancer cell types, including breast cancer cell lines MCF-7 and MDA-MB-231 (Fig. 2b and Supplementary Fig. 3b). Further, in addition to PTBP1, we also explored the possible regulation of AXL by the homologous PTBP2. As a result, we have further demonstrated that it is PTBP1, but not PTBP2, that could down-regulate AXL mRNA (Fig. 2c) and protein expression (Fig. 2d) in CL1-5 cells. As a further demonstration, we also examined the effects of knocking down the expression of PTBP1 by siRNA. As a result, up-regulation of AXL expression by PTBP1 siRNA was observed in other lung cancer cell lines, including A549 and H1299 (Fig. 2d, and Supplementary Fig. 3c). These results indicate that PTBP1 may target site-2 sequence of the AXL 5′-UTR and result in destabilization of the AXL mRNA.

### Binding of PTBP1 to AXL 5′-UTR *in vitro* and *in vivo*

**Figure 3:**
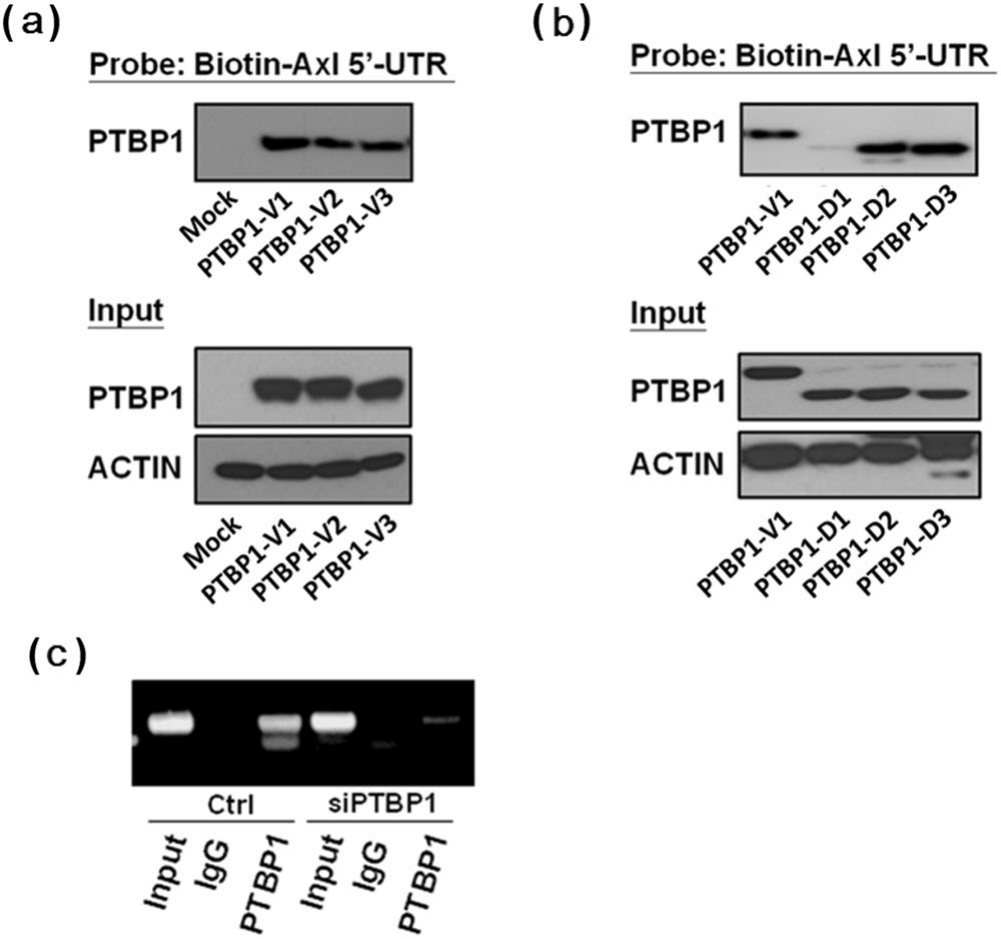
The PTBP1 protein directly binds to the AXL 5′-UTR *in vitro* (**A**,**B**) and *in vivo* (**C**). For (**A**,**B**), CL1-5 cells were respectively transfected with PTBP1 variants or various PTBP1-RRM deletion constructs. The RNA-protein binding assay was performed using biotin-labeled AXL-5′-UTR as the probe. For (**C**), The CL1-5 cells were transfected with PTBP1 siRNA and RNA-IP assay was performed as described in Materials and Methods. At least three independent experiments were performed for each assay.

In light of the fact that AXL expression is indeed regulated by PTBP1, we would like to know whether the inhibition of AXL expression by PTBP1 is achieved through its physical interaction with the AXL′s mRNA 5′-UTR. If so, which parts of PTBP1 are involved. For this purpose, we constructed expression vectors for various normal variants and various mutants of PTBP1, including deletions of different RNA recognition motifs (RRM). The *in vitro* RNA-protein binding assay was performed to examine the binding of AXL mRNA with cellular proteins of the various CL1-5 transfectants. As a result, while all three PTBP1 variants including variant 1, variant 2 and variant 3 (PTBP1-V1, PTBP1-V2 and PTBP1-V3) could interact with the biotin-labeled AXL mRNA 5′-UTR probe (Fig. 3a), PTBP1 lacking the RRM1 domain (PTBP1-D1) failed to bind the biotin-labeled AXL 5′-UTR probe (Fig. 3b). The *in vivo* RNA-immunoprecipitation (RNA-IP) assay protocol was modified from the conventional ChIP assay (chromatin immunoprecipitation) and was used to evaluate the binding of PTBP1 to the 5′-UTR of AXL mRNA when PTBP1 expression was knocked down by siRNA (Fig. 3c) and when PTBP1 or PTBP2 were ectopically overexpressed in CL1-5 cells (Supplementary Fig. 4). The results show that there is a direct binding between PTBP1 protein and the AXL 5′-UTR (Fig. 3c and Supplementary Fig. 4), demonstrating that PTBP1 may regulate AXL expression via AXL 5′-UTR targeting.

### PTBP1 regulates the stability of AXL mRNA

**Figure 4:**
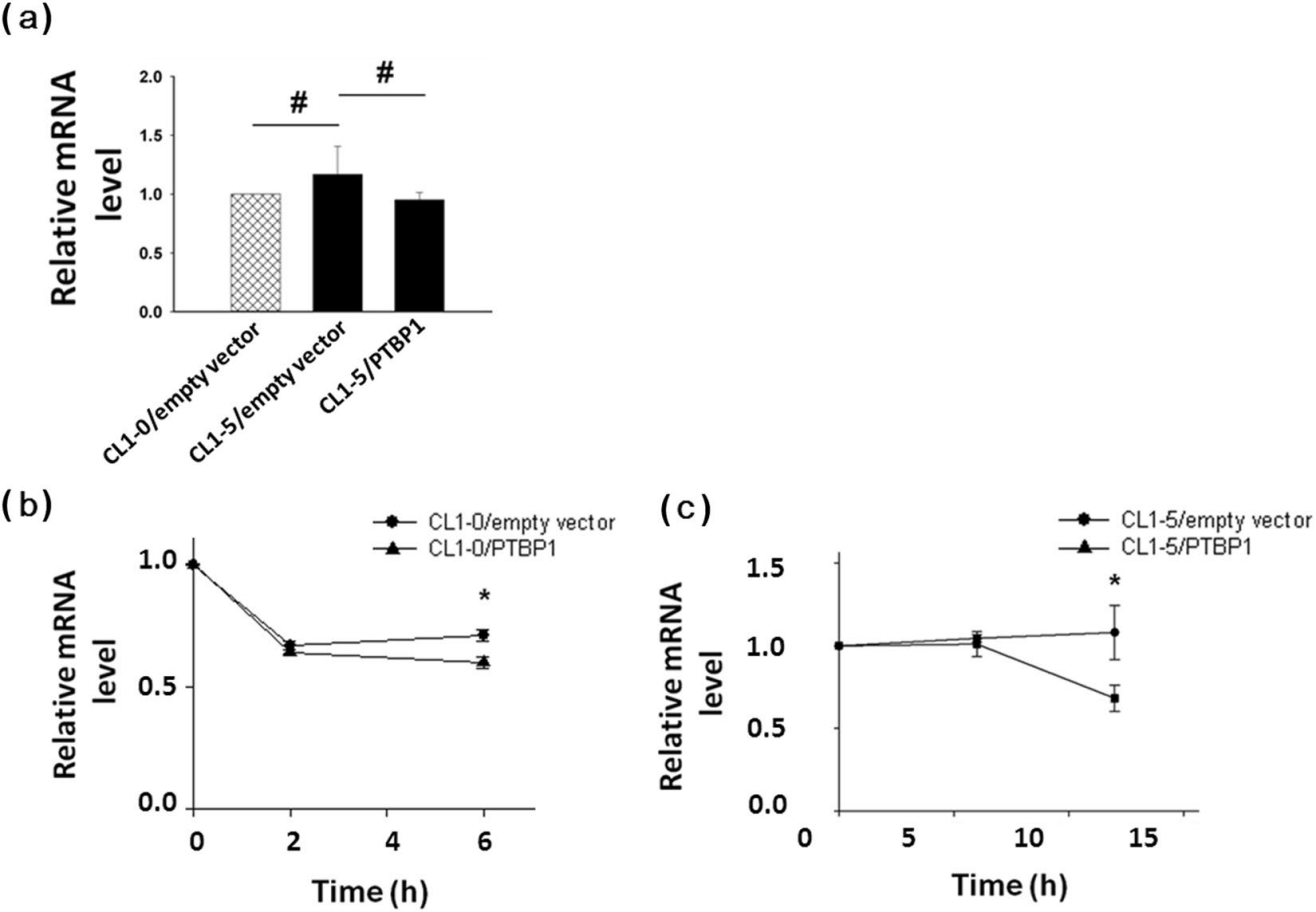
Post-transcriptional control of AXL mRNA stability by PTBP1. (**A**) The transcription rate of AXL was similar in different cells. Nuclei from 2 × 10^6^ cells were collected and incubated in a reaction buffer containing ATP, GTP, CTP and biotin-16-UTP and subjected to nonradioactive nuclear run-on assay. In some reactions (negative controls), UTP was used in place of biotin-16-UTP. Total RNA was isolated by TRIzol extraction, and biotinylated RNA was purified using agarose-conjugated streptavidin beads. Expression level of AXL mRNA was measured by quantitative real-time RT-PCR. Actin was used as the internal control. The mRNA stability measurement experiments were initiated by adding 10 μg/ml ActD to (**B**) Empty vector-transfected CL1-0 vs. PTBP1-transfected CL1-0 and to (**C**) Empty vector-transfected CL1-5 vs. PTBP1-transfected CL1-5, respectively. Cells were harvested at intervals as indicated and AXL mRNA decay was analyzed by quantitative real-time RT-PCR. The values were derived from three independent experiments. Error bars represent SD. *P < 0.05.

In this study, we have found that PTBP1 could regulate AXL mRNA expression (Fig. 2c), and its expression levels are inversely correlated with AXL mRNA levels (Fig. 2b and Supplementary Fig. 3b). To determine whether PTBP1 regulates AXL mRNA expression through affecting its transcription rates or via other post-transcriptional mechanisms, we performed the nuclear run-on assays. The differential expression of AXL mRNA in CL1-0 and CL1-5 cells is unlikely to result from altered transcription rates of the AXL gene, as examined by nuclear run-on assays with nuclei prepared from these cells. The transcription rates of AXL were neither affected by PTBP1 (Fig. 4a and Supplementary Fig. 5a). These results indicated that a post-transcriptional mechanism was involved. The AXL mRNA expression was down-regulated in the low invasive CL1-0 cells with low AXL than the highly invasive CL1-5 cells with high AXL levels (Supplementary Fig. 5b). Additionally, to determine if the AXL mRNA stability is affected by PTBP1, we examined the half-life of AXL mRNA by employing the transcription inhibitor actinomycin D in CL1-0 and CL1-5 cells with or without ectopic over-expression of PTBP1. As a result, PTBP1 was found to down-regulate the stability of AXL mRNA (Fig. 4b,c), suggesting that PTBP1 may act as a trans-acting factor regulating AXL mRNA stability.

### PTBP1 suppresses cell viability and promotes apoptosis

**Figure 5:**
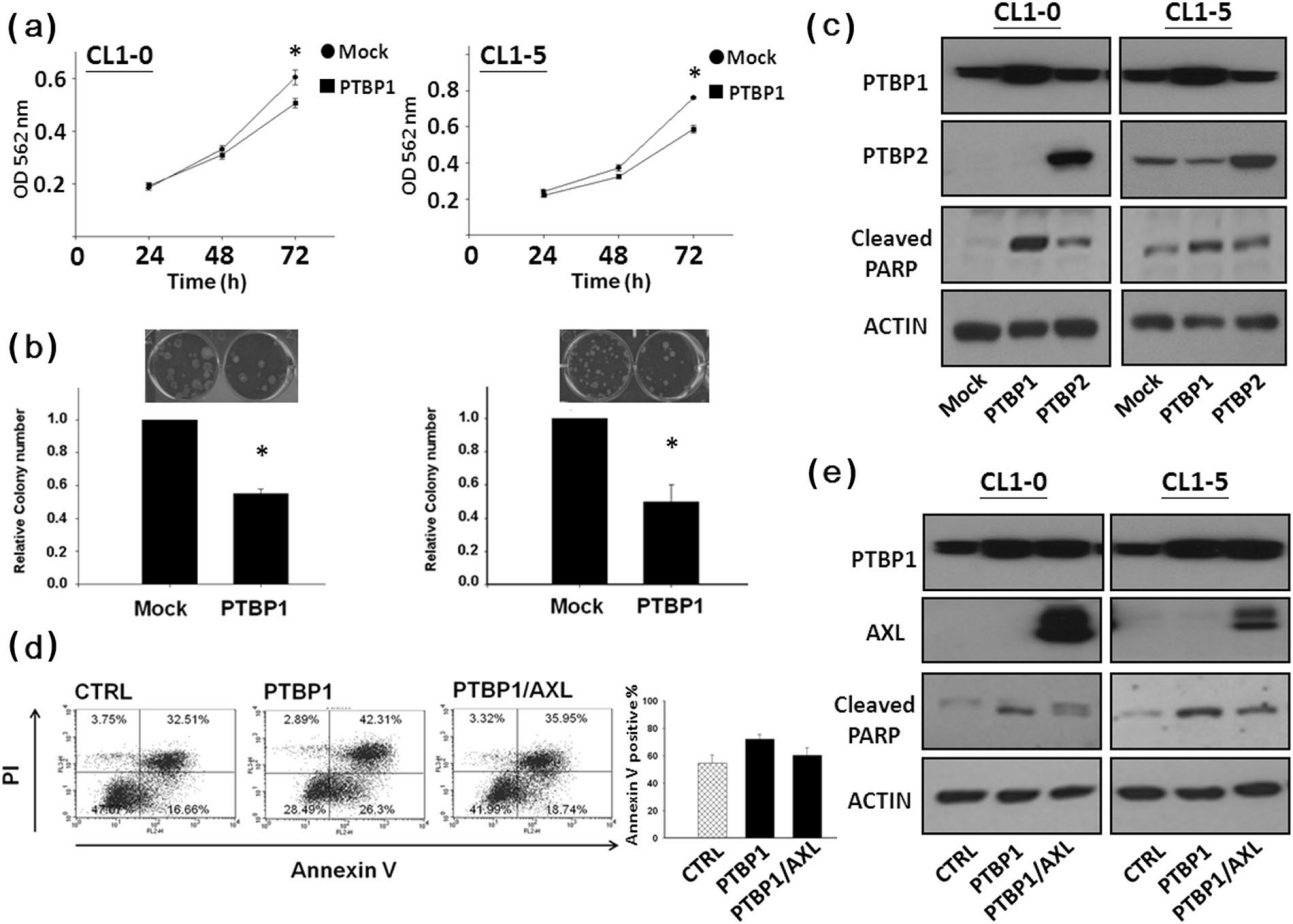
AXL expression attenuates PTBP1-mediated apoptosis. Ectopic expression of PTBP1 in CL1-0 and CL1-5 cells inhibited cellular proliferation (**A**) and colony formation (**B**). (**C**) Cellular PARP is cleaved by PTBP1 over-expression. Cells were transfected with the mock, PTBP1 or PTBP2 vector, respectively. Western blot analysis of poly (ADP-ribose) polymerase (PARP) cleavage was performed 48 h later. Whole cell lysates were subjected to Western blotting using antibody against PTBP1, PTBP2 and PARP, respectively. Actin was used as an internal control for protein loading. (**D**) Over-expression of AXL counteracts the PTBP1-mediated apoptosis. After transfection, cells were allowed to express PTBP1 for 3 days prior to harvest. Apoptosis was determined by annexin V-FITC/PI double staining followed by flow cytometry analysis. The number in the lower right quadrant indicates the percentage of early apoptotic cells. The number in the upper right quadrant indicates the percentage of late apoptotic cells, and presented as mean ± SD. (**E**) Western blot analysis of poly (ADP-ribose) polymerase (PARP) cleavage. Whole cell lysates were subjected to Western blotting using antibody against PTBP1, AXL and PARP, respectively. Actin was used as an internal control for protein loading.

Next, we looked into the PTBP1’s biological functions. MTT assay and colony formation assay were used to examine the effects of PTBP1 on cell growth and survival. The data show that over-expression of PTBP1 significantly attenuated cell growth and survival in CL1-0 and CL1-5 cells (Fig. 5a,b). In addition, while PTBP2 only slightly induced poly-ADP-ribose polymerase (PARP) cleavage, PTBP1 strongly increased PARP cleavage in both CL1-0 and CL1-5 cells (Fig. 5c). Flow cytometric analysis was used to determine if the PTBP1-mediated apoptosis was affected by AXL. CL1-0 cells were first transfected with AXL construct, the next day, cells were transfected with PTBP1 expression vector. We found that over-expression of AXL may counteracted the PTBP1-mediated apoptosis (Fig. 5d) and decreased PTBP1-mediated PARP cleavage in CL1-0 and CL1-5 cells (Fig. 5e). These results suggest that expression of AXL may attenuate PTBP1-induced apoptosis.

### PTBP1 regulates tumor growth *in vivo*

**Figure 6:**
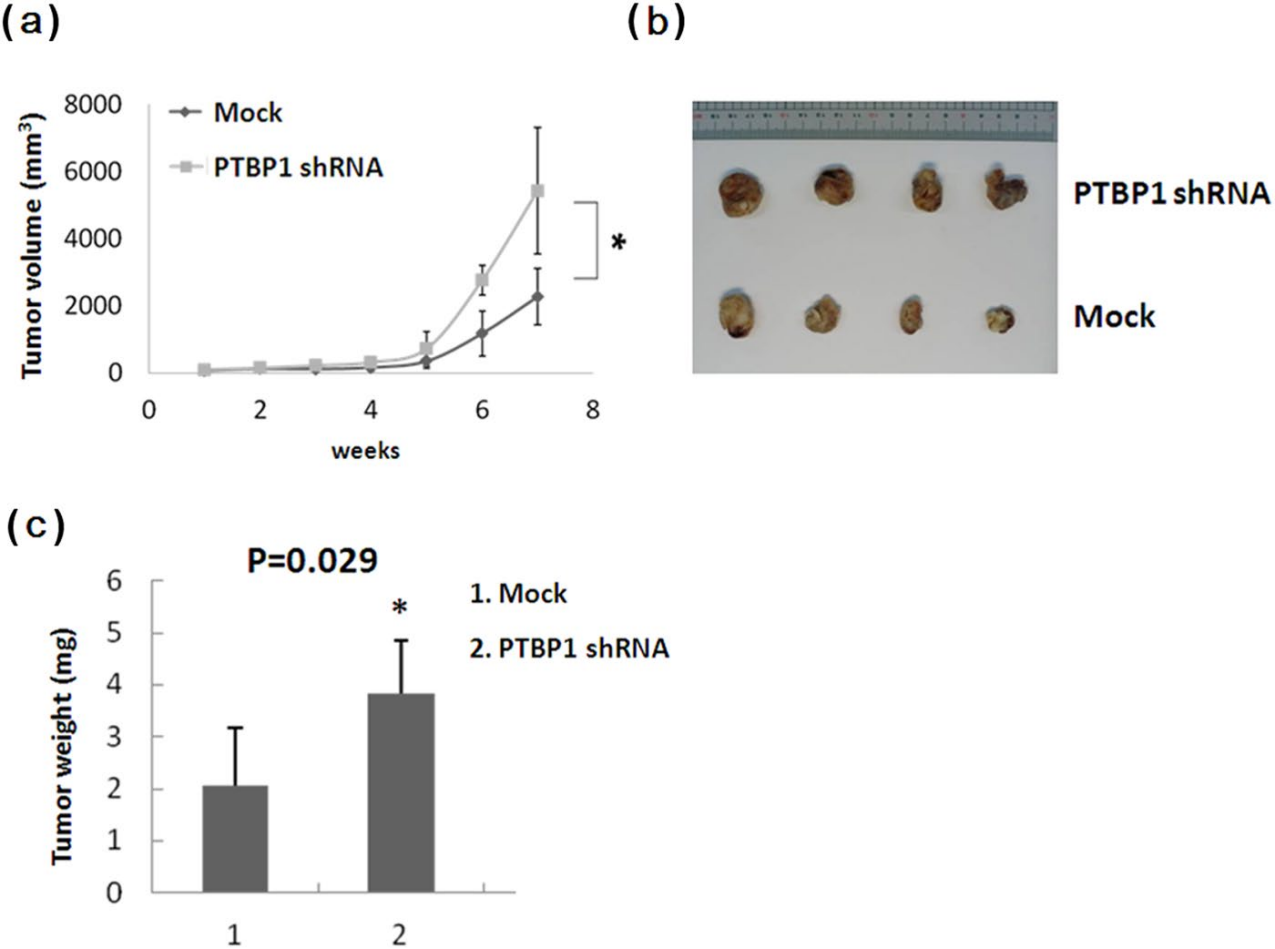
(**A**) Knockdown of PTBP1 expression enhances tumor growth. CL1-5 cells transfected with PTBP1 shRNA or control vector were implanted subcutaneously, at 1 × 10^6^ cells/mouse, into the flanks of nude mice. Tumors were observed and volumes measured up to 7 weeks post implantation. Bars represent SD. *p < 0.05. (**B**) Representative tumor nodules excised at sacrifice. (**C**) CL1-5 xenograft tumor weights. At week 7 after implantation, mice were euthanized and tumor nodules were excised, weighed, and presented as mean ± SD. *p < 0.05.

To determine the effect of PTBP1 on lung tumor growth *in vivo*, we s.c. injected nude mice with stable transfectants of shPTBP1 or mock control vector. Data show that knockdown of PTBP1 expression significantly enhanced CL1-5 tumor growth (Fig. 6a). Representative images of tumors excised from each group were shown in Fig. 6b. In agreement with the tumor growth inhibition, the tumor weights were significantly increased when PTBP1 expression was knocked down (Fig. 6c), demonstrating the tumor suppressive effect of PTBP1 *in vivo*.

### Negative correlation between PTBP1 and AXL expression in lung cancer tissues

**Figure 7:**
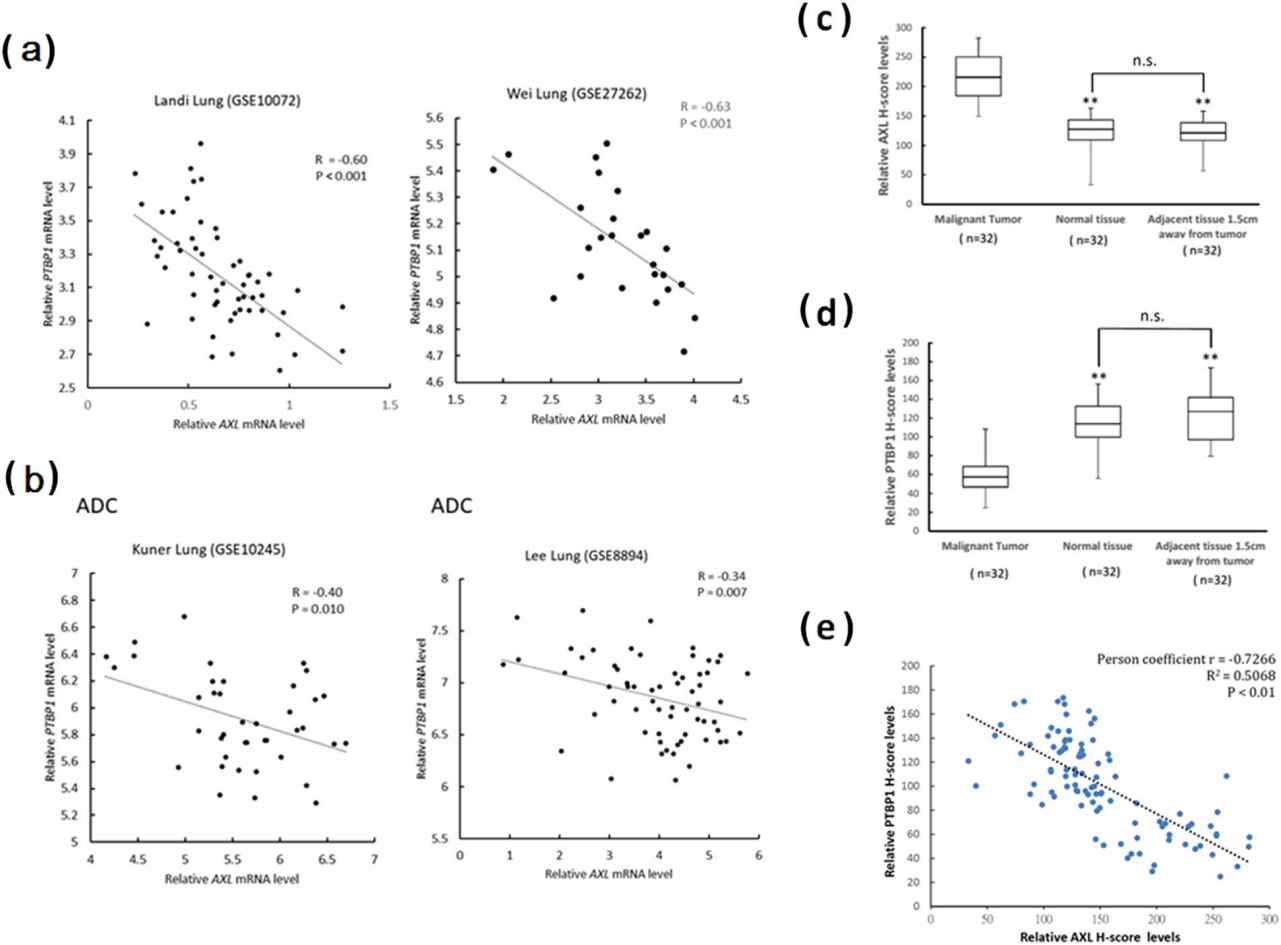
Expression levels of AXL and PTBP1 in lung tissues and tumors. Inverse correlation between expression levels of AXL and PTBP1 in both normal vs. tumor (**A**) and normal vs. adenocarcinoma (**B**) in Oncomine datasets. Graphs displaying the comparisons between the H-scores of AXL (**C**) and PTBP1 (**D**) with respect to benign (normal and adjacent tissues) and lung cancer tissues were presented. In comparison to normal tissues, the mean H-scores for AXL were significantly higher in lung cancer tissues and the mean H-scores for PTBP1 were significantly lower (*P < 0.01). (**E**) Negative correlation between AXL and PTBP1 expression. **P < 0.01.

To explore the possible clinical associations between PTBP1 and AXL, we made use of the Oncomine cancer profiling database and conducting lung tissue microarray analysis. Several lung cancer datasets, including GSE10245, GSE10072, GSE27262, and GSE8894, were analyzed and downloaded from Oncomine. The inverse correlation between expression levels of AXL and PTBP1 in normal vs. tumor (GSE10072: r = −0.60, *P* < 0.001; GSE27262: r = −0.63, *P* < 0.001) and normal vs. adenocarcinoma (ADC) (GSE10245: r = −0.40, *P* < 0.01; GSE8894: r = −0.34, *P* < 0.007) were observed as shown in Fig. 7a,b, respectively. The Oncomine datasets used in this study and the associated probes information were shown in Supplementary Table 1. Additionally, in our lung tissue microarray analysis, as shown in Supplementary Table 2, no significant differences were observed in the expression of AXL and PTBP1 (H-scores) among various clinicopathological variables examined (age, sex and pathological stage). However, AXL staining was greater in lung cancer tissues. The mean AXL score was higher in the lung cancer cohort than in the normal cohort (217.9 ± 37 vs 120.8 ± 29.9, P < 0.01) (Fig. 7c), whereas the mean PTBP1 H-score was lower in the lung cancer cohort than in the normal cohort (57.8 ± 17.8 vs 116.7 ± 22.4, P < 0.01) (Fig. 7d). PTBP1 was observed to be negatively correlated with AXL expression in lung cancer tissues (r = −0.7266, P < 0.01; Fig. 7e). These data suggest that PTBP1 and AXL may be involved in lung cancer progression.

## Discussion

AXL is associated with cancer invasion/progression, investigation of its gene regulation is of great importance, especially for its down regulation and/or inhibition in terms of clinical implications of counteracting cancer metastasis and reversing drug resistance. In this study, we have identified a molecular mechanism of AXL regulation by PTBP1 through controlling the AXL mRNA stability. We found that PTBP1, but not PTBP2, could down-regulate AXL mRNA by directly targeting its 5′-UTR. These findings may serve as an alternative way to attenuate AXL pathways as opposed to directly inhibit the AXL signaling, and may eventually help develop novel therapeutic approaches to avoid the development of cancer drug resistance and metastasis.

The splicing regulatory functions of PTBP1 have been previously correlated with malignant transformation^13–16^. While PTBP1 could shuttle between the nucleus and cytoplasm, few reports investigated its roles in cytoplasm or its differential regulatory mechanisms and functions. CD154 (the CD40 ligand) plays a role in anti-tumor activity and growth inhibitory effects in breast cancer^33,34^ and recent studies indicate that PTBP1 may stabilize CD154 mRNA via targeting its 3′-UTR^21,22^. Additionally, both our studies and others have shown that PTBP1 could down regulate AXL and HIF-1α expression in lung cancer cells via regulating their stability^25^, suggesting that PTBP1 may play roles as either a stabilizing or destabilizing factor in cytoplasm to mediate biological functions and affect cancer progression. Acting as a stabilizing or destabilizing factor may depend on the nature of the target sequences of mRNAs that PTBP1 binds and/or the cell context, i.e., involvement of other factors such as the RNA-binding protein HUR. It has previously been reported that HUR may work together with PTBP1 to regulate the translation of the HIF-1α^35^.

Recent studies indicate that overexpression of PTBP1 alone is not sufficient to cause transformation in normal cells. No significant changes in cell proliferation and anchorage-independent growth were observed in PTBP1-transfected WI-38 cells. PTBP1 overexpression alone does not stimulate cellular growth or induce a transformed phenotype *in vitro*^30^. In addition, several studies indicate that cAMP-dependent protein kinase (PKA) could phosphorylate several proteins, leading to apoptosis, cell growth arrest, and blockade of cancer cells motility and may function as a tumor suppressor^36–41^. Several tumor suppressors are known PKA downstream effectors^42–44^. Interestingly, phosphorylation of PTBP1 at Ser16 by activated PKA may stimulate its accumulation in the cytoplasm^45^. Indeed studies have shown that cancer cells have an altered PTBP1 expression^27,28^ and the RNA splicing regulatory role of PTBP1 in the nucleus plays a role in promoting malignant phenotype in cancer cells^17,29–31^. These findings indicate that PTBP1 may play different roles in the nucleus vs. cytoplasm, and hence, in normal vs. cancer cells by affecting its cellular localization. Additional supports for the hypothesis come from the fact that PTBP1 can up-regulate the tumor suppressor p27 and the Apaf-1 by binding and activating the IRES (internal ribosome entry site) of their mRNAs in cytoplasm^23,24^. Therefore, the detailed regulatory mechanism of AXL modulated by PTBP1, especially the roles of PTBP1 in the cytoplasm vs. nucleus remains to be further clarified.

The PTBP1 could down regulate HIF-1α expression via regulating its mRNA stability and was able to inhibit cell invasion when localized in the cytoplasm^25^. PTBP1 could induce p19 mRNA expression via promoter regulation and inhibit cell proliferation^26^. Deletion of the RNA recognition motif 1 (RRM1) or RRM3 of PTBP1 abolished the interaction of PTBP1 and HIF-1α mRNA. On the other hand, deletion of the RRM3 was less effective in p19 expression and cell growth inhibition, although PTBP1 may regulate p19 induction through an indirect mechanism. These results suggest that RNA-binding activity of PTBP1 is crucial in p19 regulation. We have similarly found that PTBP1 lacking of RRM1 (PTBP1-D1) failed to bind 5′-UTR of AXL mRNA *in vitro* (Fig. 3b). The RRM1 of PTBP1 is therefore crucial for this interaction in mediating biological functions and cancer progression.

The increased expression of PTBP1 may modulate pre-mature stop codon of PTBP2 resulting in nonsense-mediated mRNA decay^46^. In neuronal development, the post-transcriptional switch from PTBP1 to PTBP2 controls a widespread alternative splicing program and regulates neural precursor cell differentiation^31^. In our results, PTBP1 suppresses cell viability, promotes apoptosis (Figs. 5a, 5b and 5d) and overexpression of PTBP1, but not PTBP2, could down-regulate AXL’s mRNA (Fig. 2c) and protein expression (Fig. 2d). By contrast, while PTBP2 only slightly increased PARP cleavage, PTBP1 drastically induced PARP cleavage in both CL1-0 and CL1-5 cells (Fig. 5c). Expression of AXL leads to activation of various downstream signaling pathways involved in proliferation, inhibition of apoptosis, and therapeutic resistance^47^. To clarify whether or not AXL is one of the main mediators of PTBP1-induced apoptosis, double transfection experiments were conducted. The results show that the PTBP1 may promote apoptosis in part through inhibition of AXL (Figs. 5d and 5e). In line with these results, overexpression of PTBP1 significantly decreased AXL expression and subsequently induced apoptosis via regulating AXL stability by targeting its mRNA 5′-UTR.

Targeting various receptor tyrosine kinases has been a focus in cancer therapy and related research. However, a major limitation of these approaches is that tumor cells eventually develop resistance and AXL activation is among the mechanisms of acquired resistance to EGFR inhibition in NSCLC treatment. In our previous studies, we have found that AXL not only is crucial in cancer cells invasiveness but also may play an important role in chemo-resistance. As an alternative to developing direct AXL inhibitors, we focus on elucidating AXL’s regulatory mechanisms that may shed lights on counteracting its actions. There have been few reports on the relationship of PTBP1 and drug resistance. In studies on pancreatic cancers, PTBP1 has been shown to regulate the spicing of the pyruvate kinase (PKM) mRNA and thereby resulting in gemcitabine resistance^17^. Given that PTBP1 may destabilize AXL mRNA, it would be interesting to investigate, in cells expressing no or little PTBP1, whether AXL inhibitors may effectively inhibit cancer cells growth because of the potential likelihood of their AXL-dependency. In addition, in lung cancer cells acquiring resistance to gefitinib or erlotinib due to AXL, it is highly likely that up-regulation of PTBP1 would recover their drug sensitivity.

In this article, we clarify the molecular mechanism of AXL expression regulation by PTBP1 through controlling its mRNA stability via binding to the 5′-UTR. Indeed, over-expression of AXL may counteract the PTBP1-mediated apoptosis and knockdown of PTBP1 expression may increase tumor growth *in vivo*. In addition, PTBP1 expression was significantly higher in normal tissues and tumor-adjacent tissues compared to tumor tissues. Elucidation of the role of PTBP1 in the AXL-related malignant phenotypes may provide new molecular insights into the mechanisms underlying tumor progression and hopefully lead to new potential therapeutic modalities.

## Methods

### Cell culture and siRNA

The human breast cancer cell lines (MCF-7 and MDA-MB-231), human lung adenocarcinoma cell lines (CL1-0 and CL1-5), and the normal human lung fibroblasts (WI-38) were cultured in RPMI medium supplemented with 10% fetal bovine serum (Gibco BRL, Rockville, MD, USA), 2 μM glutamine, 100 U/ml penicillin, and 100 μg/ml streptomycin, at 37 °C in a humidified incubator with 5% CO_2_. The siRNA sequence for PTBP1: 5′-CUGUGCCUAGCAAUAUU-3′ (MDBio).

### Plasmids construction

The AXL promoter reporter and PTBP2 expression vector were constructed by insertion of the PCR-amplified sequences into the NheI/XhoI site of the pGL3 vector and EcoRI site of pcDNA3 vector, respectively. All PTBP1 variants were constructed by insertion of the PCR-amplified sequences into the BamHI/XhoI site of the pcDNA3 vector. The AXL 5′-UTR reporters were constructed in the pMIR-Report vector by inserting full-length and various mutation or deletion forms of the AXL 5′-UTR sequences into the BamHI restriction site of the pMIR-Report vector. Expression constructs of various RRM mutants of PTBP1 were as described previously^25^. All primers used for these constructs are listed in Supplemental Table 3.

### Luciferase reporter assays

Cells were transfected with a mixture of Lipofectamine 2000 (Invitrogen) and the firefly luciferase reporter plasmids pGL3-AXL promoter (−1726 ~ +1), pMIR-AXL 5′-UTR (full-length, wild-type), or various pMIR-AXL 5′-UTR mutations (deletions and putative PTBP1-binding sites mutations), along with pRL-SV40 (the Renilla luciferase transfection control). The cells were incubated for 24 h to allow expression from the plasmids and harvested using the Dual Glo luciferase assay system reagents (Promega). Luminescence was quantified using the FluoStar Optima Plate Reader (BMG Labtech, Cary, NC, USA).

### Immunoblots

Primary antibodies to AXL (amino and carboxyl terminals), PTBP1, PTBP2 and PARP1 were from Santa Cruz Biotechnology. HRP-conjugated anti-goat, anti-mouse, and anti-rabbit secondary antibodies were from Santa Cruz Biotechnology. Antibodies to Actin and GAPDH were from Sigma-Aldrich. Antibody to the PTBP1-D1 proteins was custom-made from Abcam (ab5642). Detection was performed using a horseradish peroxidase-conjugated secondary antibody and enhanced chemiluminescence (ECL Plus Kit; Amersham, Arlington Heights, IL) following the manufacturer’s instruction.

### Quantitative real-time RT-PCR

Total RNA were extracted from cells using Trizol according to the manufacturer’s instruction. The first strand of cDNA was synthesized using the Superscript III kit (Invitrogen). PCR reactions were performed in a 20 μl reaction mixture containing 10 μM forward and reverse primers, 1X SYBER GREEN reaction mix (Takara). Actin served as an internal control for loading normalization. All reactions were performed in an ABI 7500 sequence detection system. The primer pairs for AXL were: 5′-CAGCGCAGCCTGCATGT-3′ (forward), 5′-TTGGCGTTATGGGCTTCG-3′ (reverse).

### Nonradioactive nuclear run-on assay

The method was based on the incorporation of biotin-16-uridine-5′-triphosphate (biotin-16-UTP) (Sigma-Aldrich) into the nascent transcripts according to a modified protocol^48^. Total RNA was isolated with Trizol reagent (Invitrogen) and streptavidin beads (Qiagen) were used to isolate the biotin-labeled run-on RNA products. Real-time PCR and RT-PCR were performed as described above.

### Determination of AXL mRNA Stability

CL1-0 and CL1-5 cells were first transfected with PTBP1 or control vector. 24 h later, actinomycin D (ActD) was added at 10 μg/ml and incubated for various periods. Total RNA was isolated and transcripts of AXL and Actin were analyzed by quantitative real-time RT-PCR.

### Extraction of nuclear and cytoplasmic proteins and RNA protein-binding assay

Nuclear and cytoplasmic proteins were isolated using the NE-PER nuclear and cytoplasmic extraction reagents (Pierce). The RNA probes were synthesized as single strands and 5′-labeled by biotin (MDBio). Total proteins isolated from the CL1-5 cells transfected with the PTBP1 variants (or the CL1-5 cells transfected with the PTBP1 RRM deletion mutants) were incubated with biotin-labeled RNA probe and pulled down with streptavidin beads. Proteins bound to the RNA probe were analyzed by Western-blot with anti-PTBP1 antibody. The AXL RNA probe sequence: 5′-CAGCCCGGCCCTGCCCCCTCCCCCGCTGGGAGCCC-3′.

### RNA immunoprecipitation assay

Protein extracts were incubated with 5 μg of antibody against PTBP1 (Santa Cruz Biotechnology) in immunoprecipitation (IP) buffer (10 mM HEPES, pH7.9, 100 mM NaCl, 1 mM MgCl_2_, 0.1% NP-40, 2% glycerol, 1 mM DTT, and 1X protease inhibitor cocktail) at room temperature (RT) for 2 h. For the control IP reaction, mouse IgG was used in place of antibody. Then, an equal volume of protein A/G agarose beads was added to the mixture and incubated for 2 h at RT. The protein A/G agarose beads were pelleted, washed with 1 ml of IP buffer, and suspended in 0.5 ml of TRIzol Reagent. The RNA was extracted, and RT-PCR was performed with specific primers for AXL: 5′-CAGGGGTGCTGAGAAGGCG-3′ (forward), 5′-CTTTCCTCAGAAGTTGTTGGG-3′ (reverse).

### Cell proliferation MTT assay

After growth for 3 days, one-tenth volume of MTT reagent (5 mg/ml, Sigma-Aldrich) was added to the PTBP1-transfected CL1-5 cells and incubated at 37 °C for three hours when the purple color was noted, the absorbance at 562 nm was measured in a micro-titer plate reader.

### Colony formation assay

Cells transfected with mock vector or PTBP1 plasmid were seeded into six-well plates at a density of 200 cells per well and cultured for 2 weeks at 37 °C in a humidified incubator with 5% CO_2_. At harvest, the cells were washed with PBS, stained with 0.5% crystal violet for 1 minute at room temperature, and the colonies were counted.

### Apoptosis analysis by flow cytometry

Apoptosis was also determined using the Annexin V-FITC/propidium iodide (PI) detection kit from BD Pharmingen. At harvest, cells were washed in cold PBS, stained with Annexin V-FITC and PI, following the manufacturer’s instructions. The stained cells were then analyzed by flow cytometry.

### Lung cancer xenografts assay

All animal model experiments were performed in accordance with a protocol approved by the Institutional Animal Care and Use Committee of National Health Research Institutes. Nude mice of 4 to 6 weeks old were used for *in vivo* tumor xenograft growth assay. A total of 1 × 10^6^ CL1-5 cells stably overexpressing PTBP1 shRNA (or the mock vector control) suspended in 100 μl of PBS with equal volume of matrigel (BD Biosciences) were injected into the flanks of mice (n = 4), respectively. Tumor volumes were calculated using the following formula: Volume (mm^3^) = width^2^ × length. Tumors were monitored weekly and excised and weighed seven weeks after inoculation.

### Expression correlation between AXL and PTBP1 in Oncomine datasets

Several lung cancer datasets, including GSE10245, GSE10072, GSE27262, and GSE8894, were downloaded from Oncomine (http://www.oncomine.com). Information about the probes of genes of interest was downloaded from Oncomine and used in this study (Supplementary Table 1). For each dataset, gene expression data were downloaded entirely without selection. Since data from Oncomine have already been processed and normalized, they were used directly for Pearson correlation analysis.

### Lung tissue microarray (TMA) and immunohistochemistry detection

Lung tissue microarrays were obtained from a commercial supplier (LC992, US Biomax, Inc, Rockville, MD, USA). The TMA (LC992) contained biospecimen from 33 patients consisting of 32 lung adenocarcinomas tissues, 34 tumor-adjacent non-tumor tissues (1.5 cm away from tumor), and 33 normal lung tissues, with single core per each case. Specific primary antibodies against AXL and PTBP1 (1:200; Santa Cruz Biotechnology) were used for IHC with a 2-step protocol. The tissue samples were formalin fixed, paraffin embedded. Tissue array sections were mounted on the positively charged SuperFrost Plus glass slide. The tissue microarray sections were cut at 5 μm in thickness. Individual cores were 1.0 mm in diameter and US Biomax supplied the following clinicopathologic characteristics of the subjects whose tissues were on the TMA: gender, age and grade. Samples were assessed in a blinded manner by one pathologist with no knowledge of the patients’ clinicopathological data. The standard H-score (scale of 0–300) was calculated based on the staining intensity and percentage of stained cells.

### Statistical analysis

Quantitative PCR and reporter assays data are presented as mean ± SD. Differences between control and treatment groups were analyzed by Student’s t-test. All experiments were performed in triplicate and repeated at least three times and P-values < 0.05 were considered statistically significant.

## Supplementary information

SUPPLEMENTARY INFO

SUPPLEMENTARY DATASET

## References

[1] Zhang Z (2012). Activation of the AXL kinase causes resistance to EGFR-targeted therapy in lung cancer. Nature genetics.

[2] Shieh YS (2005). Expression of axl in lung adenocarcinoma and correlation with tumor progression. Neoplasia.

[3] Wimmel A, Glitz D, Kraus A, Roeder J, Schuermann M (2001). Axl receptor tyrosine kinase expression in human lung cancer cell lines correlates with cellular adhesion. Eur J Cancer.

[4] Hafizi S, Ibraimi F, Dahlback B (2005). C1-TEN is a negative regulator of the Akt/PKB signal transduction pathway and inhibits cell survival, proliferation, and migration. FASEB journal: official publication of the Federation of American Societies for Experimental Biology.

[5] Hasanbasic I, Cuerquis J, Varnum B, Blostein MD (2004). Intracellular signaling pathways involved in Gas6-Axl-mediated survival of endothelial cells. American journal of physiology. Heart and circulatory physiology.

[6] Lee WP, Wen Y, Varnum B, Hung MC (2002). Akt is required for Axl-Gas6 signaling to protect cells from E1A-mediated apoptosis. Oncogene.

[7] Fridell YW (1996). Differential activation of the Ras/extracellular-signal-regulated protein kinase pathway is responsible for the biological consequences induced by the Axl receptor tyrosine kinase. Molecular and cellular biology.

[8] Lay JD (2007). Sulfasalazine suppresses drug resistance and invasiveness of lung adenocarcinoma cells expressing AXL. Cancer research.

[9] Hong CC (2008). Receptor tyrosine kinase AXL is induced by chemotherapy drugs and overexpression of AXL confers drug resistance in acute myeloid leukemia. Cancer letters.

[10] Brand TM (2014). AXL Mediates Resistance to Cetuximab Therapy. Cancer research.

[11] Garcia-Blanco MA, Jamison SF, Sharp PA (1989). Identification and purification of a 62,000-dalton protein that binds specifically to the polypyrimidine tract of introns. Genes & development.

[12] Patton JG, Mayer SA, Tempst P, Nadal-Ginard B (1991). Characterization and molecular cloning of polypyrimidine tract-binding protein: a component of a complex necessary for pre-mRNA splicing. Genes & development.

[13] He X, Ee PL, Coon JS, Beck WT (2004). Alternative splicing of the multidrug resistance protein 1/ATP binding cassette transporter subfamily gene in ovarian cancer creates functional splice variants and is associated with increased expression of the splicing factors PTB and SRp20. Clinical cancer research: an official journal of the American Association for Cancer Research.

[14] Jin W, Bruno IG, Xie TX, Sanger LJ, Cote GJ (2003). Polypyrimidine tract-binding protein down-regulates fibroblast growth factor receptor 1 alpha-exon inclusion. Cancer research.

[15] Jin W, McCutcheon IE, Fuller GN, Huang ES, Cote GJ (2000). Fibroblast growth factor receptor-1 alpha-exon exclusion and polypyrimidine tract-binding protein in glioblastoma multiforme tumors. Cancer research.

[16] McCutcheon IE, Hentschel SJ, Fuller GN, Jin W, Cote GJ (2004). Expression of the splicing regulator polypyrimidine tract-binding protein in normal and neoplastic brain. Neuro-oncology.

[17] Calabretta S (2016). Modulation of PKM alternative splicing by PTBP1 promotes gemcitabine resistance in pancreatic cancer cells. Oncogene.

[18] Clower CV (2010). The alternative splicing repressors hnRNP A1/A2 and PTB influence pyruvate kinase isoform expression and cell metabolism. Proceedings of the National Academy of Sciences of the United States of America.

[19] David CJ, Chen M, Assanah M, Canoll P, Manley JL (2010). HnRNP proteins controlled by c-Myc deregulate pyruvate kinase mRNA splicing in cancer. Nature.

[20] He X, Arslan A D, Ho T-T, Yuan C, Stampfer M R, Beck W T (2014). Involvement of polypyrimidine tract-binding protein (PTBP1) in maintaining breast cancer cell growth and malignant properties. Oncogenesis.

[21] Coles LS (2004). A multi-protein complex containing cold shock domain (Y-box) and polypyrimidine tract binding proteins forms on the vascular endothelial growth factor mRNA. Potential role in mRNA stabilization. European journal of biochemistry / FEBS.

[22] Hamilton BJ, Genin A, Cron RQ, Rigby WF (2003). Delineation of a novel pathway that regulates CD154 (CD40 ligand) expression. Molecular and cellular biology.

[23] Cho S, Kim JH, Back SH, Jang SK (2005). Polypyrimidine tract-binding protein enhances the internal ribosomal entry site-dependent translation of p27Kip1 mRNA and modulates transition from G1 to S phase. Molecular and cellular biology.

[24] Mitchell SA, Brown EC, Coldwell MJ, Jackson RJ, Willis AE (2001). Protein factor requirements of the Apaf-1 internal ribosome entry segment: roles of polypyrimidine tract binding protein and upstream of N-ras. Molecular and cellular biology.

[25] Wang MJ, Lin S (2009). A region within the 5′-untranslated region of hypoxia-inducible factor-1alpha mRNA mediates its turnover in lung adenocarcinoma cells. The Journal of biological chemistry.

[26] Lin, S. K., Wang, M. J. & Tseng, K. Y. Polypyrimidine Tract-Binding Protein Induces p19(Ink4d) Expression and Inhibits the Proliferation of H1299 Cells. *Plos One* **8** (2013). 10.1371/journal.pone.0058227 PMC3594294 23536791

[27] Faustino NA, Cooper TA (2003). Pre-mRNA splicing and human disease. *Genes &*. development.

[28] Venables JP (2006). Unbalanced alternative splicing and its significance in cancer. BioEssays: news and reviews in molecular, cellular and developmental biology.

[29] He X (2007). Knockdown of polypyrimidine tract-binding protein suppresses ovarian tumor cell growth and invasiveness *in vitro*. Oncogene.

[30] Wang C (2008). Polypyrimidine tract-binding protein (PTB) differentially affects malignancy in a cell line-dependent manner. The Journal of biological chemistry.

[31] Cheung HC (2009). Splicing factors PTBP1 and PTBP2 promote proliferation and migration of glioma cell lines. Brain: a journal of neurology.

[32] Cho CY (2016). Negative feedback regulation of AXL by miR-34a modulates apoptosis in lung cancer cells. Rna.

[33] Gomes EM (2009). Antitumor Activity of an Oncolytic Adenoviral-CD40 Ligand (CD154) Transgene Construct in Human Breast Cancer Cells. Clinical Cancer Research.

[34] Tong AW (2001). Growth-inhibitory effects of CD40 ligand (CD154) and its endogenous expression in human breast cancer. Clinical Cancer Research.

[35] Galban S (2008). RNA-binding proteins HuR and PTB promote the translation of hypoxia-inducible factor 1alpha. Molecular and cellular biology.

[36] Chiaradonna F, Balestrieri C, Gaglio D, Vanoni M (2008). RAS and PKA pathways in cancer: new insight from transcriptional analysis. Frontiers in bioscience: a journal and virtual library.

[37] Hoelzinger DB (2005). Gene expression profile of glioblastoma multiforme invasive phenotype points to new therapeutic targets. Neoplasia.

[38] Howe AK (2004). Regulation of actin-based cell migration by cAMP/PKA. Biochimica et biophysica acta.

[39] Jiang P, Enomoto A, Takahashi M (2009). Cell biology of the movement of breast cancer cells: intracellular signalling and the actin cytoskeleton. Cancer letters.

[40] Prasad KN (2003). Defects in cAMP-pathway may initiate carcinogenesis in dividing nerve cells: a review. Apoptosis: an international journal on programmed cell death.

[41] Hafner S (1994). Mechanism of inhibition of Raf-1 by protein kinase A. Molecular and cellular biology.

[42] Collins SP, Reoma JL, Gamm DM, Uhler MD (2000). LKB1, a novel serine/threonine protein kinase and potential tumour suppressor, is phosphorylated by cAMP-dependent protein kinase (PKA) and prenylated *in vivo*. The Biochemical journal.

[43] Richter AM, Walesch SK, Wurl P, Taubert H, Dammann RH (2012). The tumor suppressor RASSF10 is upregulated upon contact inhibition and frequently epigenetically silenced in cancer. Oncogenesis.

[44] Ko FC (2013). PKA-induced dimerization of the RhoGAP DLC1 promotes its inhibition of tumorigenesis and metastasis. Nature communications.

[45] Xie J, Lee JA, Kress TL, Mowry KL, Black DL (2003). Protein kinase A phosphorylation modulates transport of the polypyrimidine tract-binding protein. Proceedings of the National Academy of Sciences of the United States of America.

[46] Spellman R, Llorian M, Smith CWJ (2007). Crossregulation and functional redundancy between the splicing regulator PTB and its paralogs nPTB and ROD1. Molecular cell.

[47] Axelrod H, Pienta KJ (2014). Axl as a mediator of cellular growth and survival. Oncotarget.

[48] Patrone G (2000). Nuclear run-on assay using biotin labeling, magnetic bead capture and analysis by fluorescence-based RT-PCR. BioTechniques.

