# Supplementary material for: PTBP1-mediated regulation of AXL mRNA stability plays a role in lung tumorigenesis
Source: Sci Rep. 2019 Nov 15;9:16922. doi: 10.1038/s41598-019-53097-2 (PMC6858377; doi:10.1038/s41598-019-53097-2)
Supplement: Supplementary file 1 — SUPPLEMENTARY INFO [file 41598_2019_53097_MOESM1_ESM.doc]

SUPPLEMENTARY FIGURES

**PTBP1-mediated regulation of AXL mRNA stability plays a role in lung tumorigenesis**

**Chun-Yu Cho^1^, Shih-Ying Chung^1^, Shankung Lin^2^, Jhy-Shrian Huang^3^, Yen-Lin Chen^4^, Shih-Sheng Jiang^1^, Li-Chun Cheng^1,5^, Tsu-Hsiang Kuo ^1,5^, Jong-Ding Lay^6^, Ya-Yu Yang^1^, Gi-Ming Lai^1,7,8^ and Shuang-En Chuang^1,*^**

^1^[National Institute of Cancer Research](http://english.nhri.org.tw/inst_cancer/index.php), National Health Research Institutes, Miaoli, Taiwan

^2^Inflammation Research & Drug Development Center, Changhua Christian Hospital, Changhua, Taiwan.

^3^Department of Pediatrics, Kaohsiung Medical University Hospital, Kaohsiung, Taiwan

^4^Department of Pathology, Cardinal Tien Hospital, School of Medicine, Fu-Jen Catholic University, New Taipei City, Taiwan

^5^Graduate Institute of Life Sciences, National Defense Medical Center, Taipei, Taiwan

^6^Department of Nursing, National Taichung University of Science and Technology, Taichung, Taiwan

^7^Comprehensive Cancer Center, Taipei Medical University, Taipei, Taiwan

^8^Cancer Center, Wan Fang Hospital, Taipei Medical University, Taipei, Taiwan.

**Supplemental figure S1:**


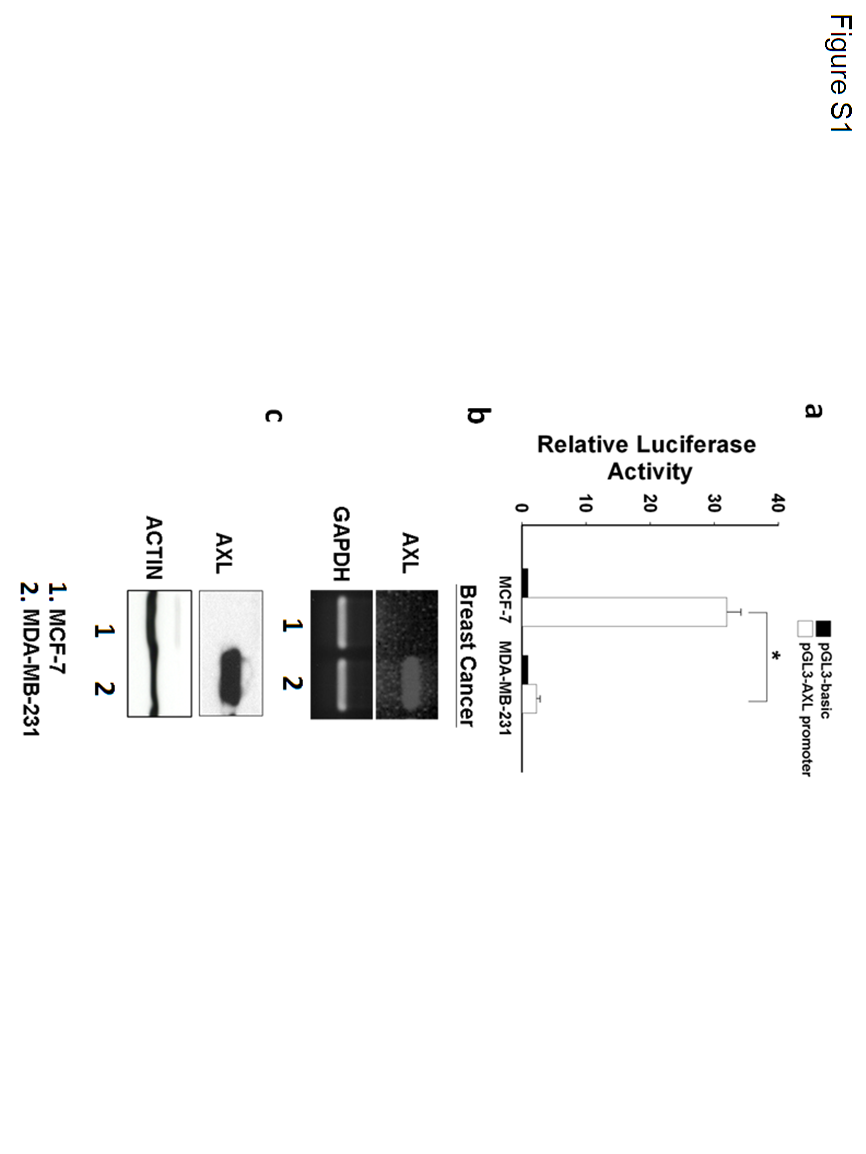


**Supp. Figure 1.** The AXL promoter activity is inversely correlated with AXL mRNA and protein expression levels in breast cancer cell lines. (a) AXL promoter reporter activity in MCF-7 and MDA-MB-231 cells. The AXL promoter activity was measured by luciferase reporter assay using breast cancer cell lines, including the less invasive MCF-7 and highly invasive MDA-MB-231. The steady state endogenous levels of AXL mRNA (b) and protein (c) of the cell lines were analyzed by RT-PCR and Western blot, respectively. GAPDH and actin were used as internal controls, respectively.

**Supplemental figure S2:**


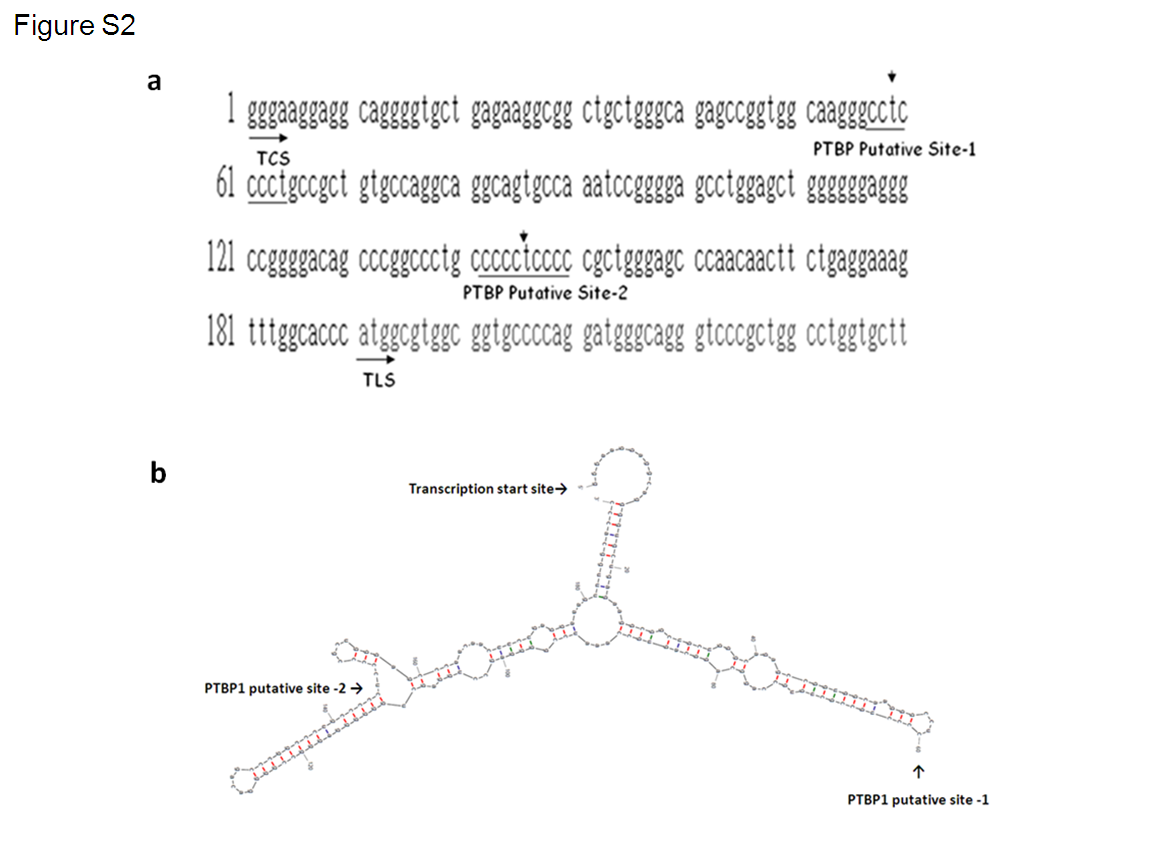


**Supp. Figure 2.** Sequence of the AXL mRNA 5’-UTR indicates two putative PTBP1 binding regions. (a) The transcription initiation site (TSS), translation start site (TLS), and potential PTBP1 binding sites are indicated and underlined. (b) The putative PTBP1 binding sites have a specific stem-loop (RNA-binding motif) structure.


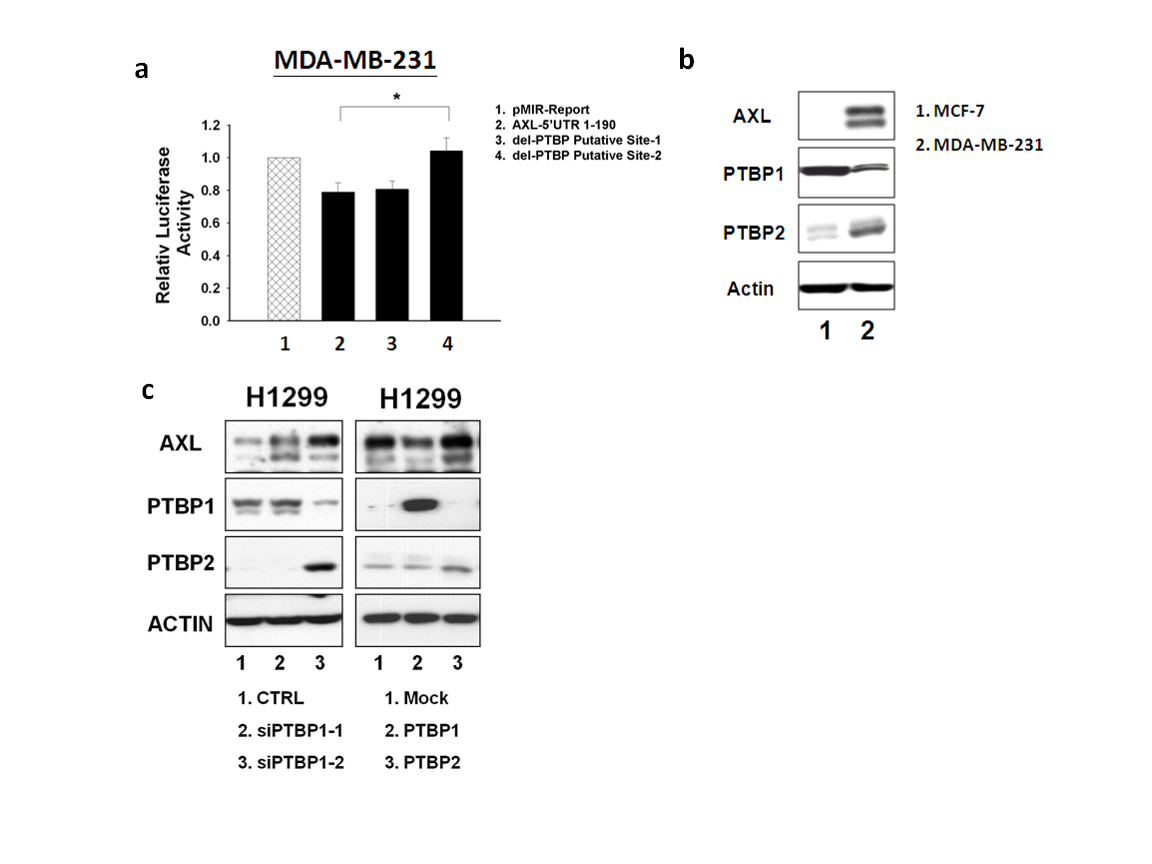
**Supplemental figure S3:**

**Supp. Figure 3.** (a) The PTBP1 putative binding site-2, but not site-1, of the AXL 5’-UTR is important for the inhibition by PTBP1 in MDA-MB-231 cells. (b) Inverse correlation between endogenous levels of AXL and PTBP1 proteins in the low invasive MCF-7 cells and the highly invasive MDA-MB-231 cells. (c) The endogenous AXL protein levels were regulated by PTBP1. H1299 cells were transfected with PTBP1, PTBP2, and siPTBP1, respectively. The protein levels of AXL, PTBP1 and PTBP2 were determined by Western blot. Actin was used as the internal control.

**Supplemental figure S4:**

**
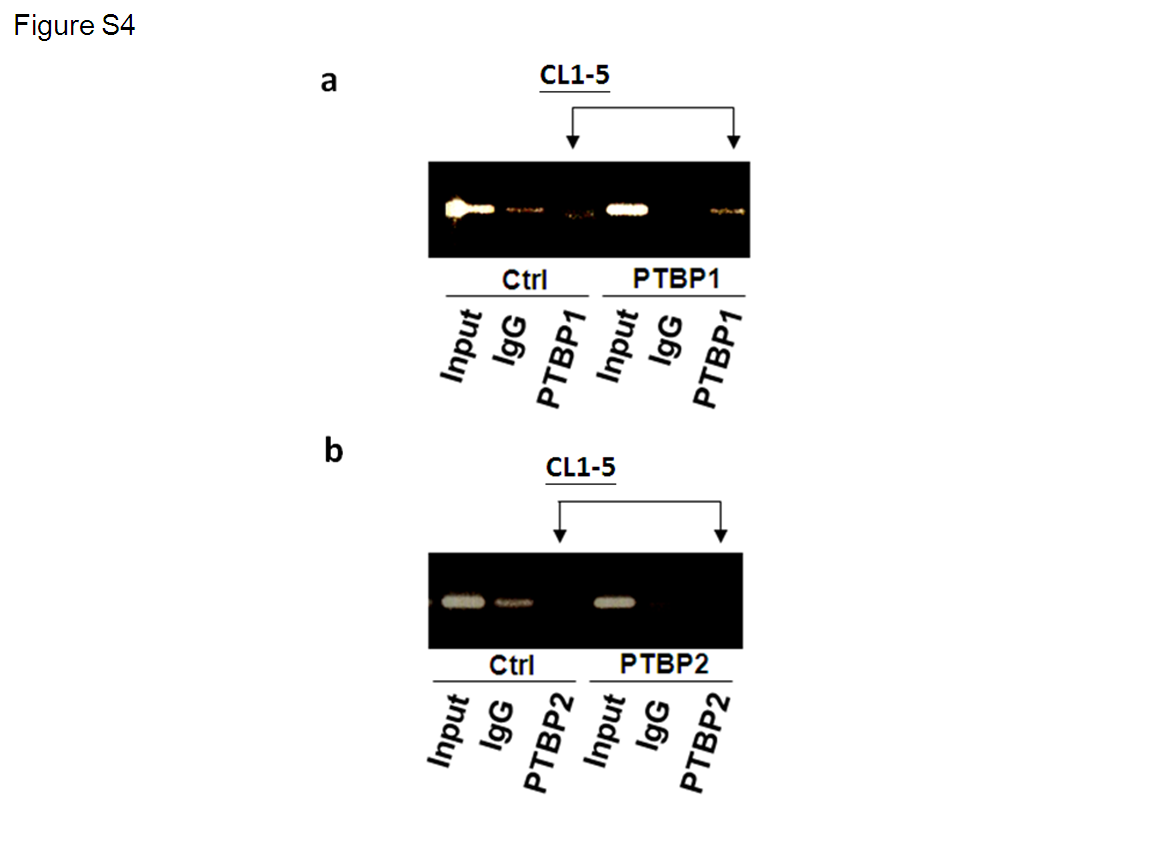
**

**Supp. Figure 4.** Binding of PTBP1 to AXL 5’-UTR *in vivo*. The PTBP1 protein directly binds to the AXL 5’-UTR as demonstrated by *in vivo* RNA-IP assay. Experiment was performed according to Nature protocol published [^49^](#_ENREF_49). Transfection of CL1-5 cells with PTBP1 up-regulated PTBP1’s binding to the AXL 5’-UTR (a). PTBP2 had no such effect (b).

**Supplemental figure S5:**

**
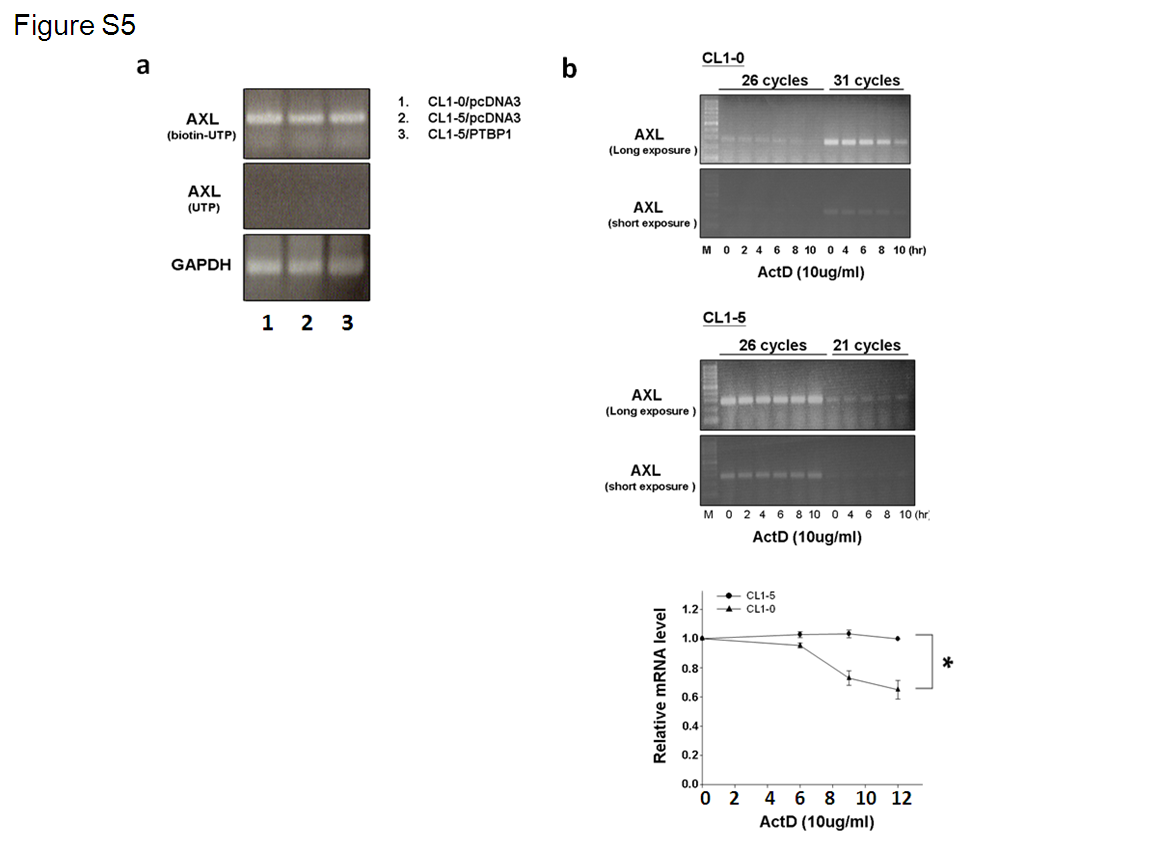
**

**Supp. Figure 5.** Post-transcriptional control of AXL mRNA stability. (a) The differential expression of AXL mRNA in cells of low vs. high invasiveness is unlikely a result of altered transcriptional rates of the AXL gene (as determined by nuclear run-on assay). (b) The mRNA half-life measurement experiments were initiated by adding 10 μg/ml ActD to CL1-0 and CL1-5 cells cultures for the intervals indicated and AXL mRNA decay was analyzed by semi-quantitative RT-PCR. For CL1-0, the number of PCR cycles were 26 and 31. For CL1-5, they were 21 and 26. The band intensity was determined by densitometer and plotted in a time-course manner.

**Supplemental figure S6:**


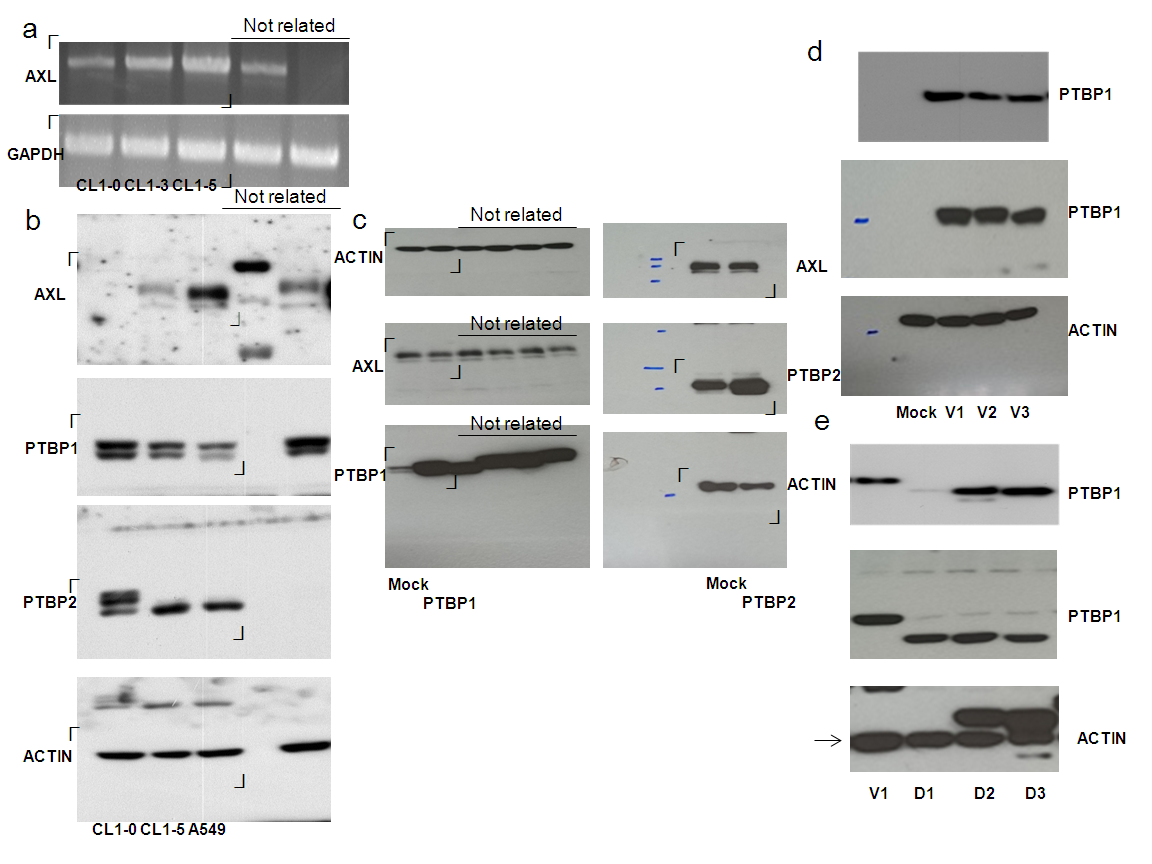


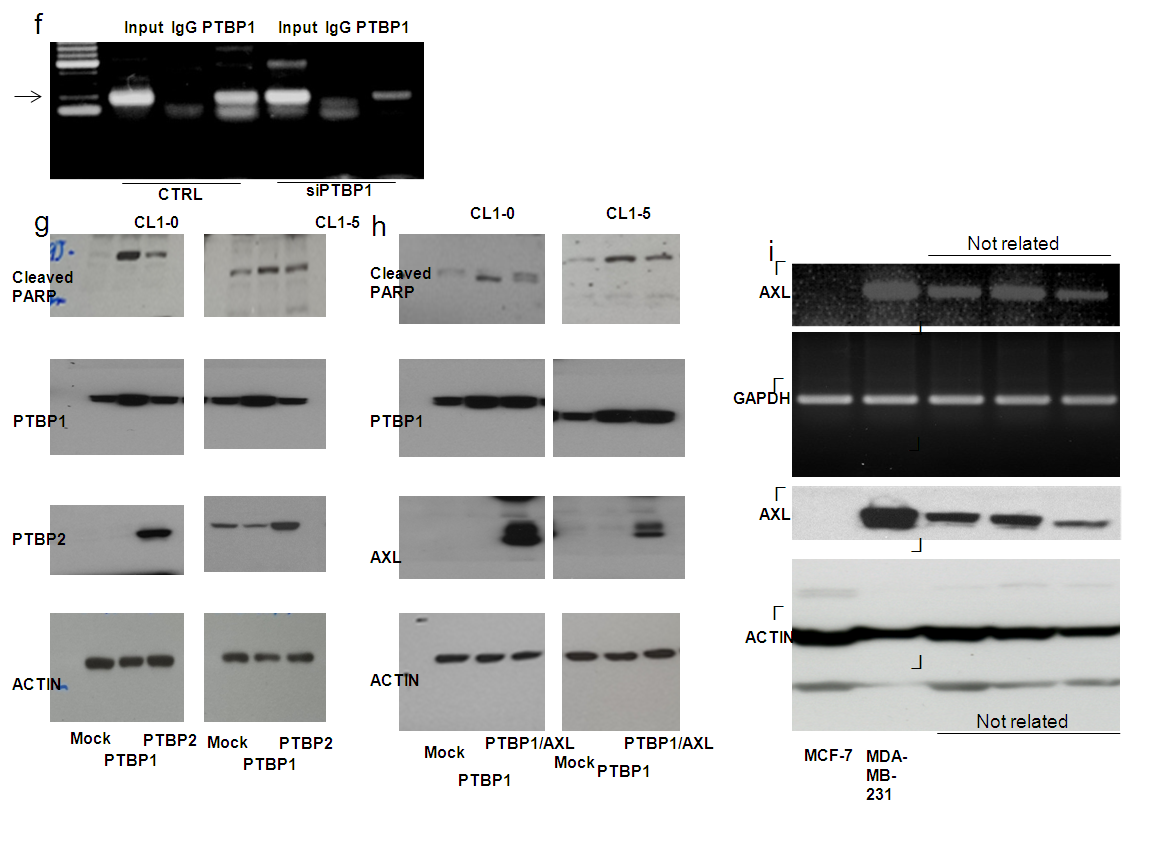


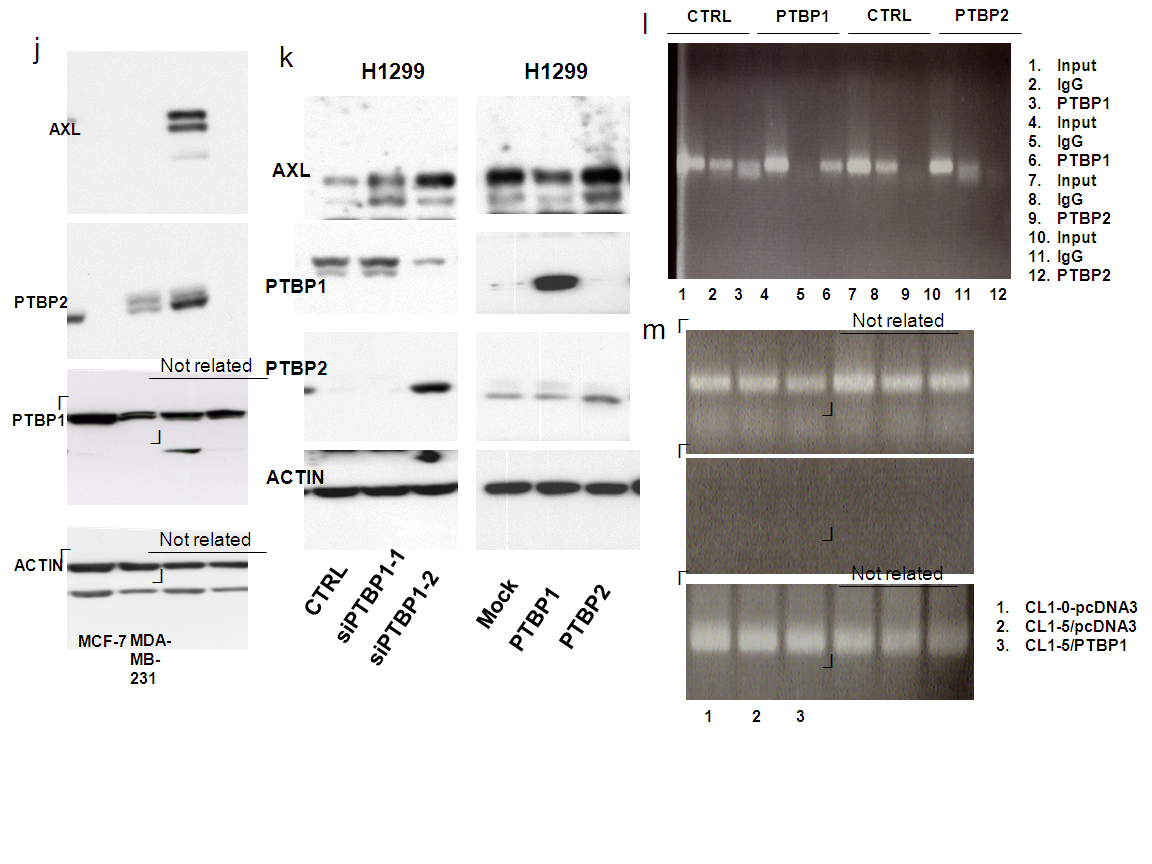


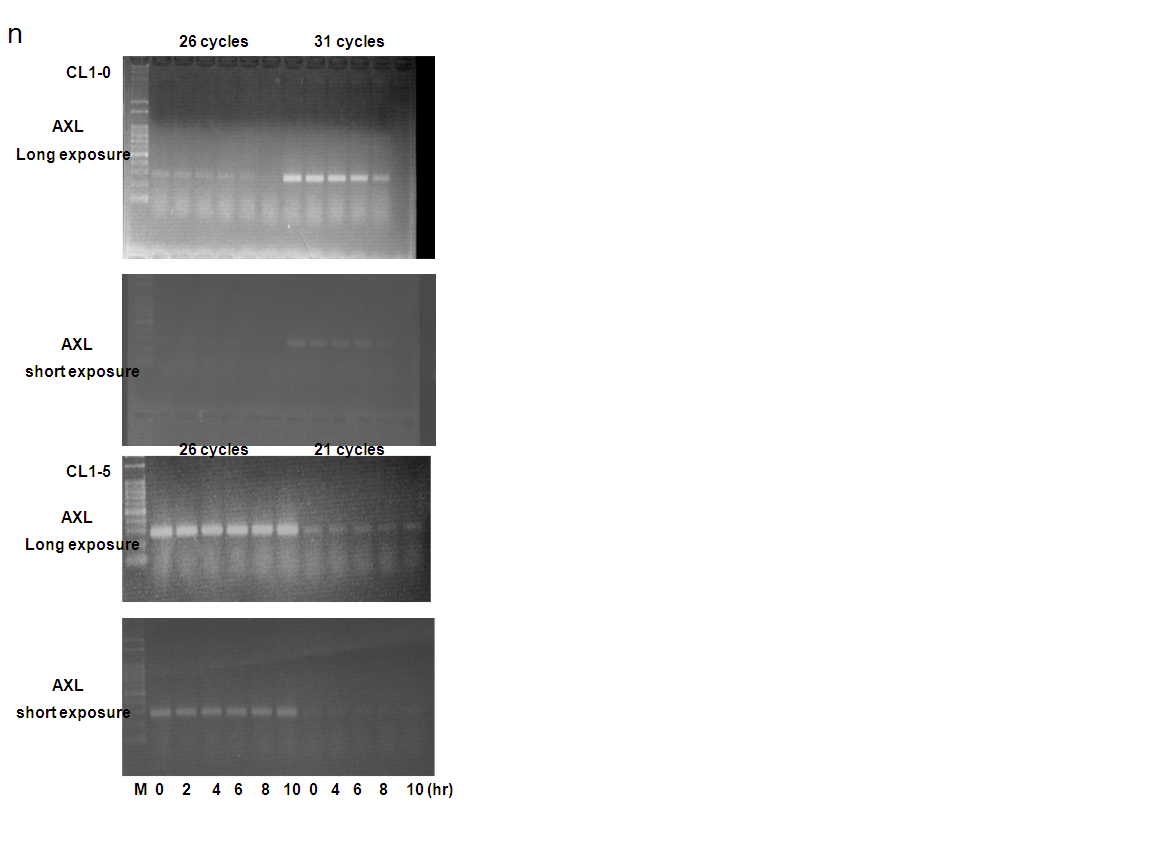


**Supp. Figure 6. Original Western blot and PCR gel images. (a) For Figure 1b.** AXL and GAPDH. **(b)** **For Figure 2b.** anti-AXL, anti-PTBP1, anti-PTBP2 and anti-ACTIN. **(c) For Figure 2d.** anti-AXL, anti-PTBP1, anti-PTBP2 and anti-ACTIN. **(d)** **For Figure 3a.** anti-AXL, anti-PTBP1 and anti-ACTIN. **(e) For Figure 3b.** anti-AXL, anti-PTBP1 and anti-ACTIN. **(f)** **For Figure 3c.** PTBP1. **(g) For Figure 5c.** anti-Cleaved PARP, anti-PTBP1, anti-PTBP2 and anti-ACTIN. **(h)** **For Figure 5e.** anti-Cleaved PARP, anti-PTBP1, anti-AXL and anti-ACTIN. **(i) For Supplemental figure S1.** anti-AXL and anti-ACTIN. **(j) For Supplemental figure S3b.** anti-AXL, anti-PTBP1, anti-PTBP2 and anti-ACTIN. **(k) For Supplemental figure S3c.** anti-AXL, anti-PTBP1, anti-PTBP2 and anti-ACTIN. **(l) For Supplemental figure S4.** AXL. **(m) For Supplemental figure S5a.** AXL and GAPDH. **(n) For Supplemental figure S5b.** AXL.

| **Supp. Table 1. Oncomine datasets used in this study and the associated probes information.** | | | | |
| --- | --- | --- | --- | --- |
| **Dataset** | **Kuner Lung (GSE10245)** | **Landi Lung (GSE10072)** | **Lee Lung (GSE8894)** | **Wei Lung (GSE27262)** |
| **Sample size** | 58 | 107 | 138 | 50 |
| **ADC** | 40 | 58 | 63 | 25 |
| **SQC** | 18 | 0 | 75 | 0 |
| **Normal** | 0 | 49 | 0 | 25 |
| **Platform** | Human Genome U133 Plus 2.0 Array (Affymetrix) | Human Genome U133 Array (Affymetrix) | Human Genome U133 Plus 2.0 Array (Affymetrix) | Human Genome U133 Plus 2.0 Array (Affymetrix) |
| **Probe ID** |  |  |  |  |
| **Axl** | 202686_s_at | 202685_s_at | 202686_s_at | 202686_s_at |
| **PTBP1** | 212015_x_at | 202189_x_at | 211270_x_at | 211270_x_at |

**Supp. Table 2. Clinicopathological characteristics of the 33 lung adenocarcinoma patients and summary of AXL and PTBP1 expression (mean H-score)**

| LC992 |  | AXL | |  | PTBP1 | |
| --- | --- | --- | --- | --- | --- | --- |
| Clnical and pathologic features | No. of patients (%) | H-score (mean ± s.d.) | P value |  | H-score (mean± s.d.) | P value |
| Age (year) |  |  |  |  |  |  |
| < 60 | 17 (53.1) | 226.7 ± 35.2 | 0.157 |  | 60.5 ± 20.3 | 0.3608 |
| ≥ 60 | 15 (46.9) | 208.0 ± 37.5 |  |  | 54.7 ± 14.5 |  |
| Sex |  |  |  |  |  |  |
| Male | 19 (59.4) | 222.8 ± 36.6 | 0.3764 |  | 56.9 ± 17.8 | 0.7384 |
| Female | 13 (40.6) | 210.8 ± 37.7 |  |  | 59.1 ± 18.4 |  |
| Grade |  |  |  |  |  |  |
| Low (I, II) | 21 (65.6) | 212.9 ± 29.3 | 0.3009 |  | 59.3 ± 20.2 | 0.5087 |
| High (III) | 11 (34.4) | 227.4 ± 48.7 |  |  | 54.8 ± 12.1 |  |

**Supp. Table 3. Primer sequences**

**AXL 5’-UTR**

**Primers**

Forward: 5’-GGGGATCCGGGAAGGAGGCAGGGGTGCT

Reverse: 5’-GGGGATCCCTGGGGCACCGCCACGCCAT

**AXL promoter**

**Primers**

Forward: 5’-CTCTGCTAGCGACACAGCCCAGGGAGACAAC

Reverse: 5’-CTCTCTCGAGGGGTGCCAAACTTTCCTCAGA

**PTBP1 variant 1 and variant 3-CDS**

Forward: 5’-GGGGATCCATGGACGGCATTGTCCCAGA

Reverse: 5’-GGCTCGAGTGGGGGCCTGTGCCCCTA

**PTBP1 variant 2-CDS**

**Primers**

Forward: 5’-GGCCGCGGCCTTCG CCTCTCCGTATGCAGGAGCT

Reverse: 5’-TGCATACGGAGAGG CGAAGGCCGCGGCCATGGTC

**PTBP2-CDS**

**Primers**

Forward: 5’-GGGAATTCTCGGCAATGGACGGAATCGTC

Reverse: 5’-GGGAATTCATCTTCCCATTTTTAAATTGTTGAC

**Del-PTBP putative site-1**

**Primers**

Forward: 5’-GCCGGTGGCAAGGGTGCCGCTGTGCCAGGCAGGC

Reverse: 5’-CTGGCACAGCGGCACCCTTGCCACCGGCTCTGCC

**Del-PTBP putative site-2**

**Primers**

Forward: 5’-CCCGGCCCTGCCCCGGGAGCCCAACAACTTCTGA

Reverse: 5’-GTTGTTGGGCTCCCGGGGCAGGGCCGGGCTGTCC
